# 2D nanomaterial sensing array using machine learning for differential profiling of pathogenic microbial taxonomic identification

**DOI:** 10.1007/s00604-022-05368-5

**Published:** 2022-07-06

**Authors:** Zhijun Li, Yizhou Jiang, Shihuan Tang, Haixia Zou, Wentao Wang, Guangpei Qi, Hongbo Zhang, Kun Jin, Yuhe Wang, Hong Chen, Liyuan Zhang, Xiangmeng Qu

**Affiliations:** 1 Key Laboratory of Sensing Technology and Biomedical Instruments of Guangdong Province, School of Biomedical Engineering, Sun Yat-Sen University, Shenzhen, 518017 China; 2 Department of Clinical Laboratory, The Seventh Affiliated Hospital of Sun Yat-Sen University, Shenzhen, 518017 Guangdong China; 3 Pharmaceutical Sciences Laboratory, Åbo Akademi University, 20520 Turku, Finland; 4 Turku Bioscience Centre, University of Turku and Åbo Akademi University, 20520 Turku, Finland; 5 School of Petroleum Engineering, State Key Laboratory of Heavy Oil Processing, China University of Petroleum (East China), Qingdao, 266580 China; 6 Pen-Tung Sah Institute of Micro-Nano Science and Technology, Xiamen University, Xiamen, 361005 China; 7 Harvard John A. Paulson School of Engineering and Applied Sciences, Harvard University, Cambridge, MA 02138 USA

**Keywords:** Pathogenic microbial taxonomic, Molecular response differential profiling, Machine learning approach, Accurate recognition

## Abstract

An integrated custom cross-response sensing array has been developed combining the algorithm module’s visible machine learning approach for rapid and accurate pathogenic microbial taxonomic identification. The diversified cross-response sensing array consists of two-dimensional nanomaterial (2D-n) with fluorescently labeled single-stranded DNA (ssDNA) as sensing elements to extract a set of differential response profiles for each pathogenic microorganism. By altering the 2D-n and different ssDNA with different sequences, we can form multiple sensing elements. While interacting with microorganisms, the competition between ssDNA and 2D-n leads to the release of ssDNA from 2D-n. The signals are generated from binding force driven by the exfoliation of either ssDNA or 2D-n from the microorganisms. Thus, the signal is distinguished from different ssDNA and 2D-n combinations, differentiating the extracted information and visualizing the recognition process. Fluorescent signals collected from each sensing element at the wavelength around 520 nm are applied to generate a fingerprint. As a proof of concept, we demonstrate that a six-sensing array enables rapid and accurate pathogenic microbial taxonomic identification, including the drug-resistant microorganisms, under a data size of *n* = 288. We precisely identify microbial with an overall accuracy of 97.9%, which overcomes the big data dependence for identifying recurrent patterns in conventional methods. For each microorganism, the detection concentration is 10^5^ ~ 10^8^ CFU/mL for *Escherichia coli*, 10^2^ ~ 10^7^ CFU/mL for *E. coli-β*, 10^3^ ~ 10^8^ CFU/mL for *Staphylococcus aureus*, 10^3^ ~ 10^7^ CFU/mL for MRSA, 10^2^ ~ 10^8^ CFU/mL for *Pseudomonas aeruginosa*, 10^3^ ~ 10^8^ CFU/mL for *Enterococcus faecalis*, 10^2^ ~ 10^8^ CFU/mL for *Klebsiella pneumoniae*, and 10^3^ ~ 10^8^ CFU/mL for *Candida albicans*. Combining the visible machine learning approach, this sensing array provides strategies for precision pathogenic microbial taxonomic identification.

**Graphical abstract:**

• A molecular response differential profiling (MRDP) was established based on custom cross-response sensor array for rapid and accurate recognition and phenotyping common pathogenic microorganism.

• Differential response profiling of pathogenic microorganism is derived from the competitive response capacity of 6 sensing elements of the sensor array. Each of these sensing elements’ performance has competitive reaction with the microorganism.

• MRDP was applied to LDA algorithm and resulted in the classification of 8 microorganisms.

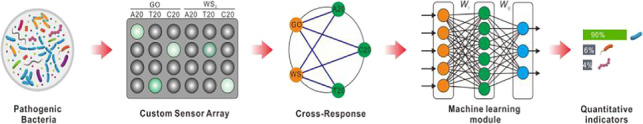

**Supplementary Information:**

The online version contains supplementary material available at 10.1007/s00604-022-05368-5.

## Introduction

Pathogenic microorganisms have rich varied, diverse surface morphology and complex biochemical characteristics, which essentially threaten human health and cause social panic upon their infection [1–4]. Antibiotics that are applied to treat pathogenic microorganism infection have been overused, leading to the thriving of antibiotic-resistance microorganisms [5–8]. To reduce the dose of the antibiotics, the accurate recognition of microbial taxonomic for multiple microorganism recognition is essential to precisely guide the medical therapy [5, 7, 9].

Some traditional methods for identifying microorganisms have been developed, including the morphological recognition method [10–12], the immunodiagnostic method [13, 14], and the molecular diagnostics method [15–17]. However, these methods require expensive reagents, instruments, higher operating skills, and low throughput, which limit the application of these methods in clinical practice. In recent years, a variety of sensing array has been developed, targeting to fulfill the requirement of multiple target microorganism detection. For example, Yan et al. have reported a fluorescence sensing array for identified five different bacteria, which six types of metal ion-protein-AuNC as sensing elements [18]; Fan et al. reported a GO-antimicrobial peptide (AMP) sensing array for identified 13 different bacteria [19]. Wu et al. have reported a sensing array of different thiopropionic acid, thiosuccinic acid, cysteine, and CTAB-functionalized AuNPs for identified 15 microorganisms [20]. These sensing arrays collect features from multiple dimensions to differentiate information for multiple target detection, including different receptors on the membrane of bacteria [21], the interaction between bacteria and sensing elements [20], and the specific metabolites [22, 23]. Combining the machine learning approach, a large amount of data can be processed for multi-target recognition. However, the kinds of sensing elements in these sensing arrays are limited, with the confined ability for the special kind of microorganism identification. Therefore, it is challenging to develop a sensing array that is not limited by the number and kinds, enabling the visualization of the detection progress.

Herein, we establish a molecular response differential profiling cross-response sensing array for rapid and accurate recognition of microorganisms. The sensing elements are composed of a series of 6-carboxyfluorescein (FAM)-labeled single-strand DNA (FAM-ssDNA) and two-dimensional nanomaterial (2D-n) fluorescence quencher. In particular, we hypothesize that non-specific competitive reactions of pathogenic microorganisms with ssDNA molecules and 2D-n build a chemical-responsive information identification method for the pathogenic microorganisms. The sensing element’s silhouette coefficient directly presents the degree of influence on the classification results of each sensing element, ensuring the approach’s identification process is visible. The cross-responsive sensing array produces a unique response differential profiling for the pathogenic microorganisms. Combining the advantage of the machine learning algorithm, we can visibly discriminate each pathogenic microorganism by regulating the species and quantity of sensing elements with 100% accuracy.

## Experimental section

### Materials

All oligonucleotide sequences were synthesized and purified by Sangon Biotech Co., Ltd. (Shanghai, China), and the specific sequence information is shown in Table S1. Graphene oxide (GO) dispersion (sheet diameter 50 ~ 200 nm) and tungsten disulfide (WS_2_) dispersion (sheet diameter 20 ~ 500 nm) were purchased by Nanjing XFNANO Material Technology Co., Ltd. (Nanjing, China). All the strains were purchased from Shanghai Luwei Technology Co., Ltd. (Shanghai, China), and the specific names and numbers are shown in Table S2. Tryptone soy broth (TSB) medium was purchased from Beijing Solarbio Science & Technology Co., Ltd. (Beijing, China); yeast extract peptone dextrose (YPD) medium was purchased from Guangdong HuanKai Microbial Technology Co., Ltd. (Guangdong, China); and buffer PBS was purchased from Sangon Biotech Co., Ltd. (Shanghai, China). Unless otherwise stated, all the aqueous solutions were prepared using deionized water and purified using a Milli-Q water purification system (Millipore Corp., Bedford, MA) with a resistivity of 18.2 MΩ/cm. Artificial urine (pH 6.0) was purchased from Beijing Leagene Biotechnology Co. Ltd. (Beijing, China). Substitution of human serum (TBDTM-HS0704) was purchased from Haoyang Biological Products Technology Co., LTD (Tianjin, China).

### Instrumentation

Microorganisms were cultured in a constant temperature incubator shaker (IS-RDV1, Crystal Technology & Industries, Inc., Dallas, TX, USA) at 37 °C with a shaking speed of 80 rpm. Autoclave manufacturer is GI54DW (Zealway Instrument Inc., Xiamen, China). Sterile operation worked in a clean bench CJ-1S (Taisite Instrument, Tianjin, China). A microplate reader (BioTek Synergy 4) was used for recording fluorescence spectrum and intensity by *λ*_ex_ = 488 nm and *λ*_em_ = 520 nm. Zeta potential and dynamic light scattering (DLS) were recorded using Malvern Zetasizer Nano ZS90.

## Methods

### Microorganism culture

Tryptone soy broth powder of 30 g was dissolved in 1 L deionized water. Yeast extract peptone dextrose of 49 g was dissolved in 1 L deionized water, stirring, heating, or else boiling until it is completely dissolved; then, it is divided into triangular bottles and autoclaved at 121 °C for 20 min and stored at 4 °C, maintaining sterility [24, 25]. Methicillin-resistant *Staphylococcus aureus*, *S. aureus*, *Enterococcus faecalism*, *Escherichia coli*, β-lactam-resistant *E. coli*, *Klebsiella pneumonia*, and *Pseudomonas aeruginosa* were cultured in a TSB medium at 37 °C and *Candida albicans* in a YPD medium at 28 °C. ATCC ID was listed in Table S2. Microorganism suspensions were measured by a microplate reader and stopped culturing by centrifuged (7500 rpm, 5 min), when they proliferated up to OD_600_ = 0.6, which is approximately equal to 1 × 10^8^ CFU·mL^−1^. Then, liquid was removed and sediment was resuspended (i.e. microorganism) in equal volume by 1 × PBS [24, 26].

### Preparation of sensing array

To construct the sensor array, DNA elements (sequences were listed in Table S1) and two-dimensional nanomaterial (GO and WS_2_) are diluted by phosphate-buffered saline (PBS), serum, and urine, respectively. The final concentrations of DNA elements and 2D-n are 10 nM and 40 μg/mL. After 12 h of incubation in a temperature room, 50 µL DNA elements and 2D-n solutions are transferred to the black 96-well plate (Sangon, China). One milliliter of the incubated DNA elements and 2D-n was transferred in a zeta potential sample cell for potential measurement [27].

### Fluorescence experiment

Eight microorganisms were diluted (10 times successively) into 10^2^–10^8^ CFU/mL, respectively. After 12 h of incubation in PBS, serum, and urine, 50 μL of DNA elements and 2D-n solutions are transferred to the black 96-well plate. At the excitation light of 488 nm at 25 °C, the fluorescence intensity at 520 nm was recorded with a microplate reader. Then, 50 μL of microorganism solutions of different densities was added to each well and incubated for a period of time. The fluorescence intensity at 520 nm was measured again, and the fluorescence difference between the two measurements was used as the fluorescence response [27].

### Statistical analysis

The fluorescence difference between the two measurements is used as the fluorescence response. Calculate (Δ*I* = *I* − *I*_0_), where *I* is the fluorescence intensity after adding microorganism, and *I*_0_ is the fluorescence intensity before adding microorganism, then normalized it by the maximum *I* value of all the datasets (abovementioned initial data are shown in Figure S1). Linear discriminant analysis (LDA) processes the fluorescence intensity data matrices to distinguish them in R (version 3.5.2). The data graphs were drawn using Origin 2020 and GraphPad Prism 8 [28].

The fitting curve of bacterial concentration and its LDA score as well as the Stern–Volmer plot and quenching efficiency plot of FAM-T20, FAM-A20, and FAM-C20 at different GO concentrations is analyzed using GraphPad Prism 8 (GraphPad, https://www.graphpad.com/guides/prism/8/user-guide/).

### Apparent recovery experiment

The array sensing was mixed with different concentrations of microorganism in serum and urine. LDA classification of different concentrations of microorganism was determined. According to the LD1 of LDA, the concentrations of microorganism in different body fluids were calculated by fitting the curve of Figure S6. The average of LD1 can be denoted as “Detected”, “Add amount,” and “Detected” which are listed in Tables S7–S8 [29, 30].

## Results and discussion

The recognition principle of the molecular response differential profiling based on the custom cross-response sensing array**.**

**Scheme 1:**
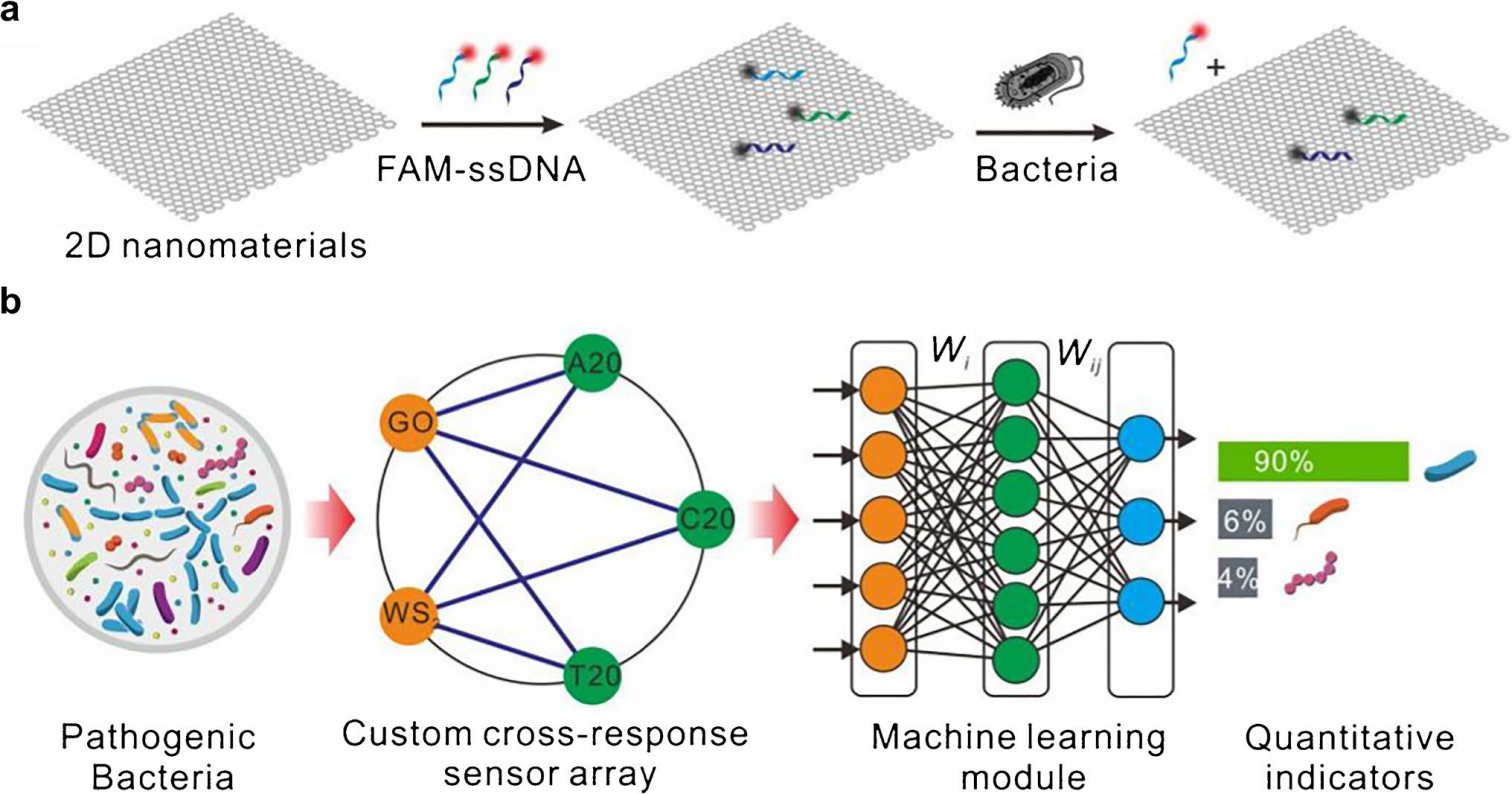
The recognition principle of molecular response differential profiling based on the custom cross-response sensing array. (a) The sensing array consisting of different FAM-labeled ssDNA (A20, T20, and C20) complexes and two different 2D nanomaterials (GO and WS_2_) fluorescence quenchers and the key steps for recognition of microbial taxonomic. (b) The chart flow of recognition of microbial taxonomic by the sensing array and machine learning. *W*_*i*_ and *W*_*ij*_ are the weight factors

In this work, we establish a series of custom sensing elements for featured extraction modulation. The sensing array’s sensing elements are composed of anionic FAM-labeled ssDNA and two types of 2D-n fluorescence quenchers, as shown in Scheme 1. The anionic FAM-labeled ssDNA binds strongly with 2D-n, resulting in fluorescence quenching due to electron transfer [27, 31–33]. Upon incubation with microorganisms, microorganisms compete with FAM-labeled ssDNA for nanoscale 2D-n binding due to the sugars, phosphates, and lipids in the outer membrane of microorganisms, which can form hydrogen bonds with 2D-n [34–36]. As such, the FAM-labeled ssDNA is displaced from the 2D-n surface, leading to the recovery of the fluorescent signal (Scheme 1a). The detected fluorescent signal intensity strongly correlates to the affinity between the 2D-n and microorganisms based on the species and quantity of sensing elements and surface physicochemical features of the microorganisms, resulting in the generation of a unique fluorescence response differential profiling to profile microorganisms (Scheme 1b).

In this work, two different 2D-n, graphene-oxide (GO) and tungsten disulfide (WS_2_) fluorescence quenchers [27, 33, 37], with FAM-labeled ssDNA (three different sequences, FAM-A20, FAM-C20, and FAM-T20) as the sensing elements’ model are chosen for perceptual differences of microorganism. To precisely visualize the fluorescence response differential profiling to profile microorganisms, a data-driven machine learning approach is applied. Taking the advantage of DNA molecules’ moldability and 2D-n’s diversity as a feature extraction layer of target microorganism, the precision visualization with machine learning can be achieved with very low data input. The diverse sensing array generates specific multiple parallel parameters, which fully connects machine learning algorithms. These machine learning algorithms convert the fluorescence response levels into low-dimensional characteristic vectors through weight matrices of sensing elements. Then, the low-dimensional characteristic vectors can be used in the evaluation of microorganism phenotyping indicators (Scheme 1b) [38].

### The feasibility research of sensing elements to convert the target pathogenic microorganisms into chemical-response identifying information

**Fig. 1:**
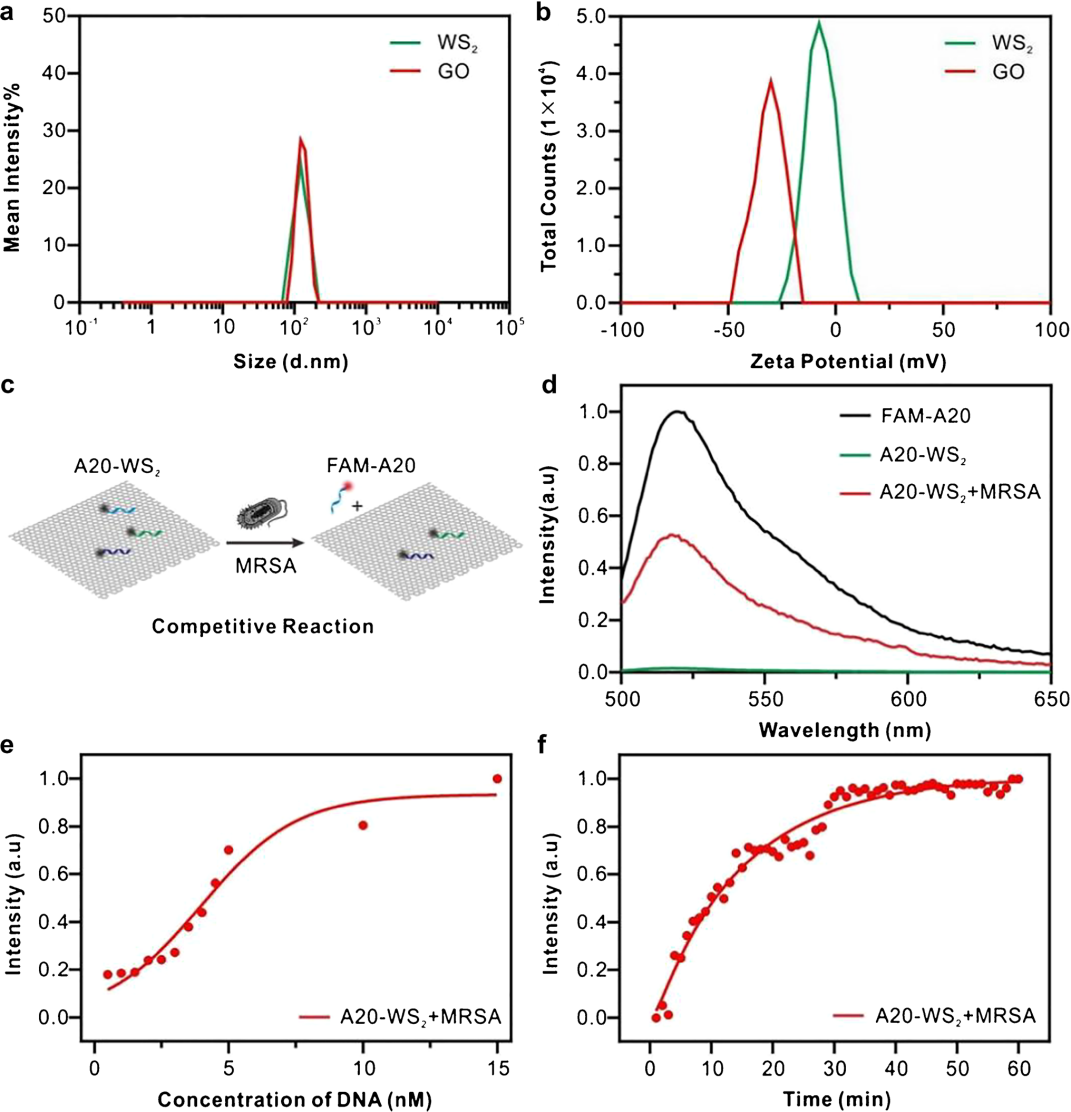
Sensing elements’ feasibility to convert the target pathogenic microorganisms into chemical-response identifying information. DLS (**a**) and zeta potential (**b**) characterization of GO and WS_2_. (**c**) Schematic representation of competitive reaction between sensing element and microorganisms. (**d**) The fluorescence signal of free FAM-A20 (10 nM, black curve), hybridization with WS_2_ conjugates (40 μg/mL, green curve), or in the presence of MRSA (6 × 10^8^ CFU/mL, red curve). (**e**) Fluorescence titration measurement of different concentrations of FAM-A20 (2 nM, 3 nM, 4 nM, 5 nM, 6 nM, 7 nM, 8 nM, 9 nM, 10 nM, 20 nM, and 30 nM, respectively) with a constant concentration of WS_2_ (40 μg/mL) in the presence of MRSA (6 × 10.^8^ CFU/mL). (**f**) Fluorescence recovery of A20-WS_2_ by time in the presence of MRSA

We systematically study the competitive reaction mechanism of microorganisms and sensing elements. Firstly, we characterize the GO and WS_2_ using DLS and zeta potential. The result shows that nanoscale GO and WS_2_ have quite uniform diameters and elevated zeta potential, as shown in Fig. 1a and b. Then, we further study the quenching process of 2D-n to FAM-DNA and the Stern–Volmer plot of the quenching ability of WS_2_ to FAM-DNA (A20/T20/C20), as shown in Figure S1. The result shows that each Stern–Volmer plot is not linear, and the curve tends to the Y-axis when WS_2_ concentration is high. In brief, the slope of the curve increases as WS_2_ concentration increases; this result indicates that the quenching system of WS_2_ and FAM-DNA exists in both static quenching and dynamic quenching. This result is consistent with the previous research [39]. This complex quenching type can be analyzed by the following formula:

$${F}_{0}/F=\left(1+{K}_{D}\left[Q\right]\right)\left(1+{K}_{S}\left[Q\right]\right)$$

$${F}_{0}/F=1+{K}_{app}\left[Q\right]$$

$${K}_{app}=\left[{F}_{0}/F-1\right]/\left[Q\right]=\left({K}_{D}+{K}_{S}\right)+{K}_{D}{K}_{S}\left[Q\right]$$

where, *F*_0_ and *F* are the fluorescence intensity before and after quenching agent is added; *K*_*app*_ is the apparent quenching constant; *K*_*D*_ and *K*_*S*_ are the dynamic and static quenching constants; and [*Q*] is the concentration of the quench agent. *K*_*app*_ or (*F*_0_/*F* − 1)/[*Q*] and [*Q*] generate a line with an intercept of *K*_*D*_ + *K*_*S*_ and a slope of *K*_*D*_*K*_*S*_ [40].

Furthermore, fluorescence quenching efficiency of FAM-T20, FAM-A20, and FAM-C20 with different concentrations of WS_2_ is shown in Figure S2. The results showed that the fluorescence intensity of FAM-DNA decreased significantly with the increase of WS_2_ concentration, and the quenching efficiency of WS_2_ for the three FAM-DNA is close to 100% when WS_2_ concentration > 30 mg/mL. The results indicate that WS_2_ has a good quenching effect on FAM-DNA.

Then, we develop a WS_2_-loaded FAM-A20 sensing element and carry out the feasibility assay to confirm our sensing element’s capability of chemical-response on pathogenic microorganisms, as shown in Fig. 1c. The fluorescence of FAM-A20 is almost completely quenched in the presence of WS_2_ due to the strong affinity of FAM-A20 to WS_2_, with quenching efficiency up to 99%, as shown in the black and green curves in Fig. 1d. When adding MRSA, the FAM-A20 exfoliates from the surface of WS_2_. Thus, the fluorescence is partially recovered, as shown in Fig. 1d, represented by the red curve. Furthermore, we quantitatively analyze the competitive reaction of MRSA and FAM-A20 to the WS_2_ in the presence of MRSA. We determine the critical concentration for quenching the fluorescent signal by adding different concentrations of FAM-A20 to a constant concentration of WS_2_ in the presence of MRSA through a titration approach, as shown in Fig. 1e. By taking FAM-A20 concentration that induces half-maximal fluorescent intensity change [41], we can calculate the apparent dissociation constant of 4.43 nM for the FAM-A20 in the competitive interaction between MRSA and WS_2_. Moreover, the competitive reaction can complete under 40 min as non-fluorescent intensity variation, as shown in Fig. 1f. Our sensing elements’ feasibility is converted from the target pathogenic microorganism binding into readable chemical-responsive information through the above quantitative analysis of the competitive reaction process.

### Specific response differential profiling analysis for microorganisms

**Fig. 2:**
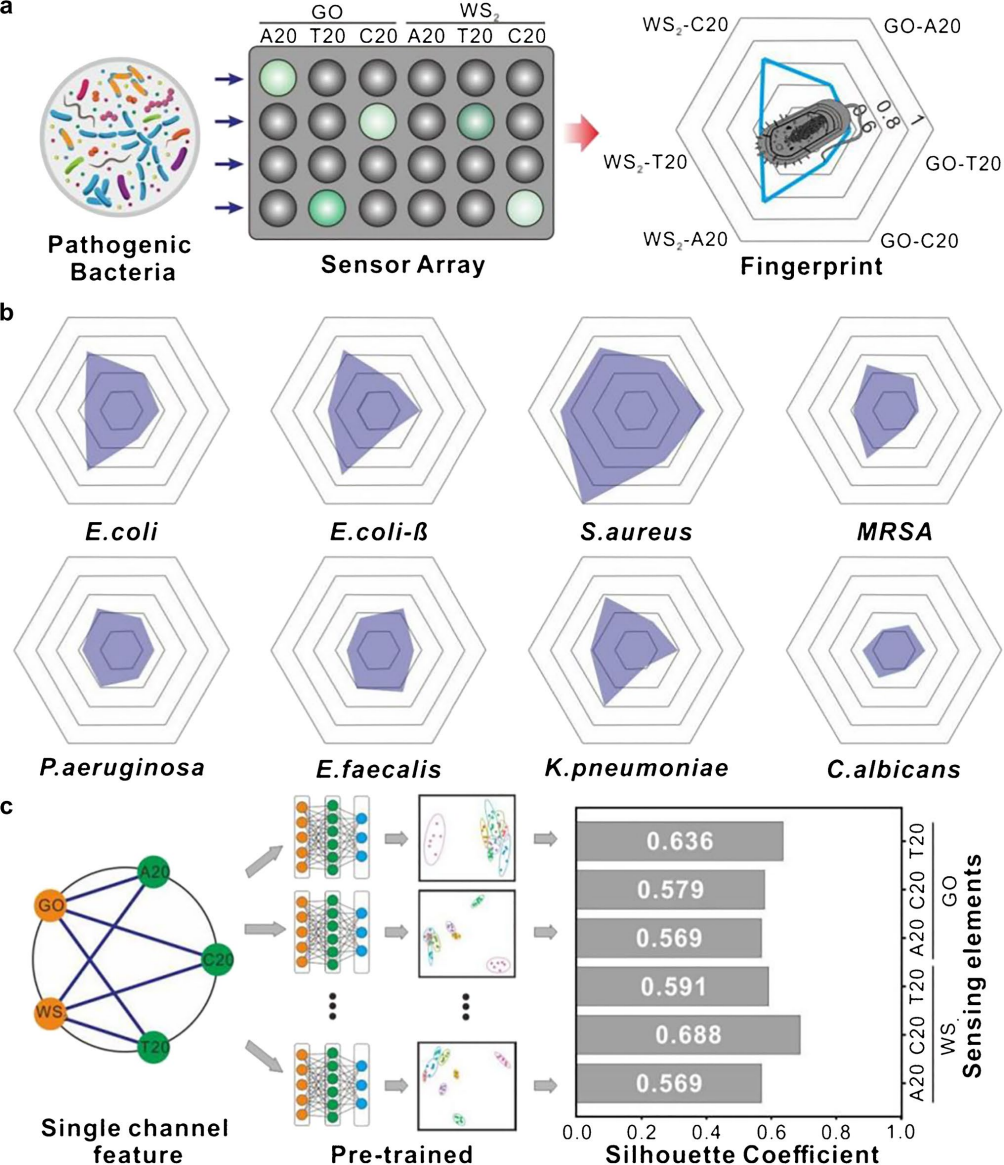
Specific response differential profiling analysis of the cross-response sensing array to target microorganisms. (**a**) The fluorescence fingerprints of different bacteria, as measured by sensing array. (**b**) Fluorescence response levels of six sensing elements (A20-GO, T20-GO, C20-GO, A20-WS_2_, T20-WS_2_, and C20-WS_2_) for different bacteria. (**c**) The response sensitivity of single sensing element by silhouette coefficient

A series of sensing elements act as custom feature extraction modules for microbial profiling. The sensing array is carried out in a 96-well microplate, and the sequence of the sensing array platform is shown in Fig. 2a. On each plate, a set of the fluorescence intensities of the initial (*I*_0_(a.u.)) and final (*I*(a.u.)) response are measured with a microplate reader in the absence and presence of microorganisms, respectively (Figure S3). We can obtain a set of the fluorescent incremental of each microbial fluorescence response through the equation of Δ*I* = *I* − *I*_0_. In this work, eight different microorganisms, including *E. coli*, β-lactam-resistant *E. coli* (*E. coli*-β), *S. aureus*, methicillin-resistant *S. aureus* (MRSA), *P. aeruginosa*, *E. faecalis*, *K. pneumoniae*, and *C. albicans* are selected as model pathogenic microorganisms. After the addition of these bacteria into the 96-well plates with preloaded 2D-n and FAM-labeled ssDNA, apparent changes in fluorescence are detected with different intensities.

Each pathogenic microorganism shows a different fluorescence response level to each of the six sensing elements (A20-NGO, T20-NGO, C20-NGO, A20-WS_2_, T20-WS_2_, and C20-WS_2_), as shown in Fig. 2b. The pathogenic microorganism’s response profile is derived from the different competitive capacities of varying sensing elements to various microorganisms. This result demonstrates the capability of the sensing array in turning target bacteria into the unique fluorescence responsive differential profiling.

The response sensibility of the single sensing element to identify microorganisms is estimated using silhouette coefficient [42, 43], which represents the similarity between clusters. The silhouette coefficient ranges from − 1 to + 1, where a high value indicates that the point is well matched to its cluster [42]. Figure 2c shows that each sensing element as a single-channel feature extraction layer can obtain a fluorescence response value for the target pathogenic microorganism. The fluorescence response value is as input information used for the pre-training of machine learning algorithms. The silhouette coefficients of T20-GO, C20-GO, A20-GO, T20-WS_2_, C20-WS_2_, and A20-WS_2_ are measured to be 0.636, 0.579, 0.569, 0.591, 0.688, and 0.569, respectively. The result indicates that our sensing array’s sensing element has good response sensitivity to identify target microorganisms and gives rise to the high silhouette coefficient. What is more, the sensing element’s silhouette coefficient visualization shows each of the sensing element’s degrees of influence on the identification results, which ensures the visibility of the machine learning approach–basic identification process.

### Identification performance analysis of the molecular response differential profiling based on the custom cross-response sensing array

**Fig. 3:**
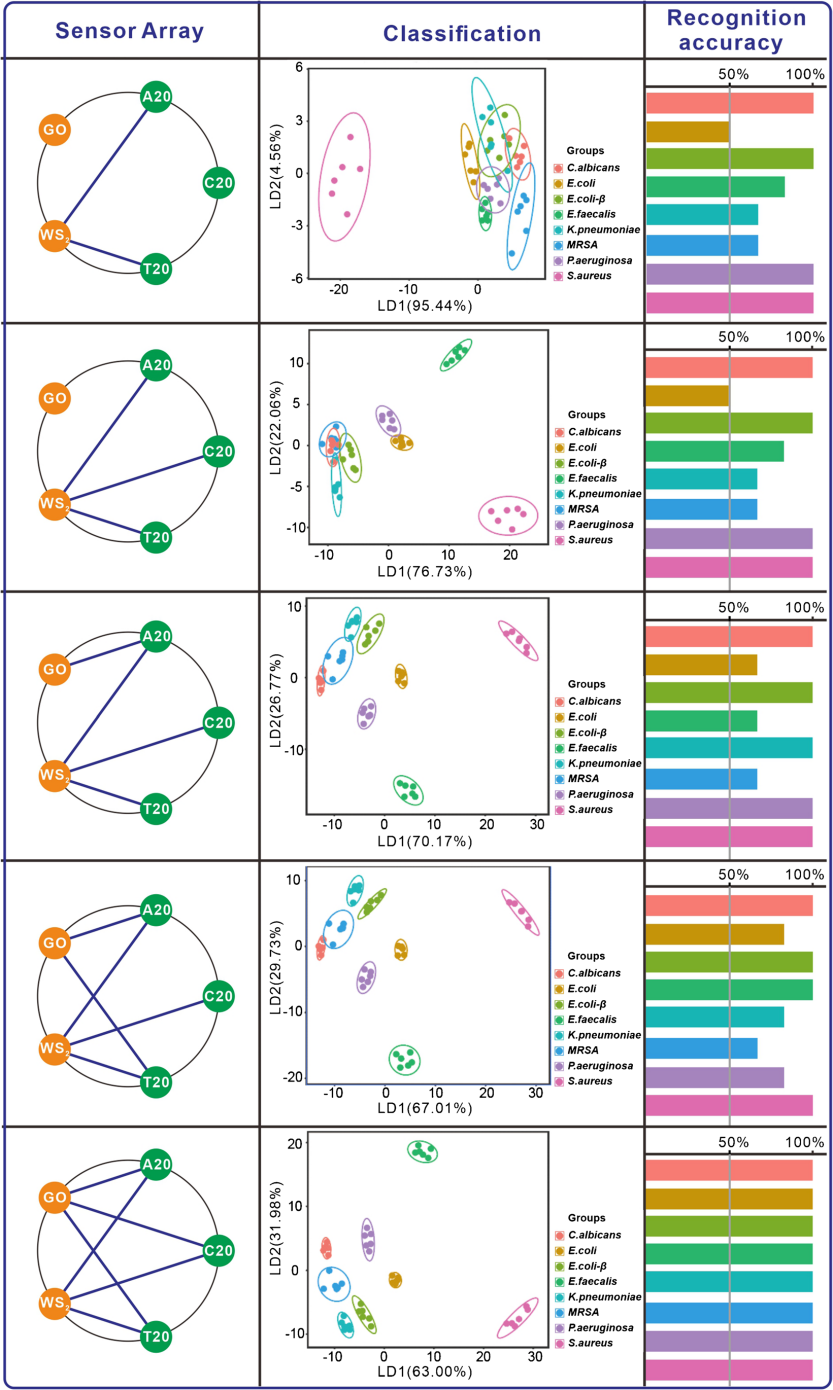
The machine learning approach’s recognition accuracy is critically dependent upon input heterogeneity. (Sensing array) The input heterogeneity from the custom sensing array. The LDA plot (Classification) and the accuracy (Recognition accuracy) of the custom sensing array’s discriminant analysis

We also systematically investigate the effects of the custom sensing array on identification accuracy. Linear discriminant analysis (LDA) [28] is used to statistically characterize the fluorescent incremental (Δ*I*). The specific adjustment programs of sensing elements, as shown in in Fig. 3 and Figure S4, the ellipsoids in Fig. 3 represent the confidence interval at 95%. First, we utilize two types of sensing elements (T20-WS_2_ and A20-WS_2_) fluorescence quenchers to recognize eight different microorganisms, and this finalizes the training matrix with 96 data points from 48 test cases (2 sensing elements × 8 microorganisms × 6 replicates), which produces linear discrimination factors of 95.44 and 4.56, and the overall recognition accuracy is 83.3%. The result shows substantial overlap among different microbial strains, especially among *E. coli*, *E. coli*-β, *E. faecalis*, *K. pneumoniae*, and MRSA by two sensing elements (T20-WS_2_ and A20-WS_2_), as shown in the first row of Fig. 3, There is a partial overlap among *E. coli*-β, *E. faecalis*, *K. pneumoniae*, and MRSA for three sensing elements (T20-WS_2_, A20-WS_2_, and C20-WS_2_). This finalized the training matrix with 144 data points from 48 test cases (3 sensing elements × 8 microorganisms × 6 replicates), which produces linear discrimination factors of 76.73 and 22.06, and the overall recognition accuracy is 83.3%, as in the second row of Fig. 3. Here, overlaps are detected among *E. coli*, *E. faecalis*, MRSA for four sensing elements (A20-GO, T20-WS_2_, A20-WS_2_, and C20-WS_2_), and this finalized the training matrix with 192 data points from 48 test cases (4 sensing elements × 8 microorganisms × 6 replicates), which produces linear discrimination factors of 70.17 and 26.77, and the overall recognition accuracy is 87.5%, as shown in the third row of Fig. 3. Furthermore, overlap among *E. coli*, *P. aeruginosa*, *K. pneumoniae*, and MRSA are detected for the five sensing elements (T20-GO, A20-GO, T20-WS_2_, A20-WS_2_, and C20-WS_2_), and this finalized the training matrix with 240 data points from 48 test cases (5 sensing elements × 8 bacteria × 6 replicates) which produces linear discrimination factors of 67.01 and 29.73, and the overall recognition accuracy is 89.6%, as shown in the fourth row of Fig. 3. Finally, these eight microorganisms are well separated when combining all six sensing elements, as shown in the fifth row of Fig. 3, including the drug-resistant microorganisms. The finalized training matrix with 288 data points from 48 test cases (6 sensing elements × 8 microorganisms × 6 replicates) produces linear discrimination factors of 63 and 31.98, and the overall recognition accuracy is 97.9%.

Furthermore, we study the classification performance of sensing array quantitatively. In brief, three main classification performance indicators are evaluated, which are Precision (the proportion of real classes in the sample predicted to be positive), Recall (the proportion predicted to be positive classes in all positive classes), and F1 score (harmonic average of accuracy rate and recall rate). The specific values of each performance indicator are as shown in Table S5. According to these data, we found that the classification ability of sensing array increases with the increase of the number of sensing elements. These results show that identification performance can be programmed by simply regulating species and quantities of sensing elements. The result confirmed that the diversity of DNA molecules and two-dimensional materials breaks through the limitation of the number and type of array sensing elements and improves the classification performance. Hence, our study reveals that the diversified sensing elements ensure extract information differentiation and the recognition data volume of the target, which overcome the dependence on big data from parallel experiments.

### Discrimination of multiple pathogenic microorganism in body fluids and determination of the Gram-status of the pathogenic microorganisms

**Fig. 4:**
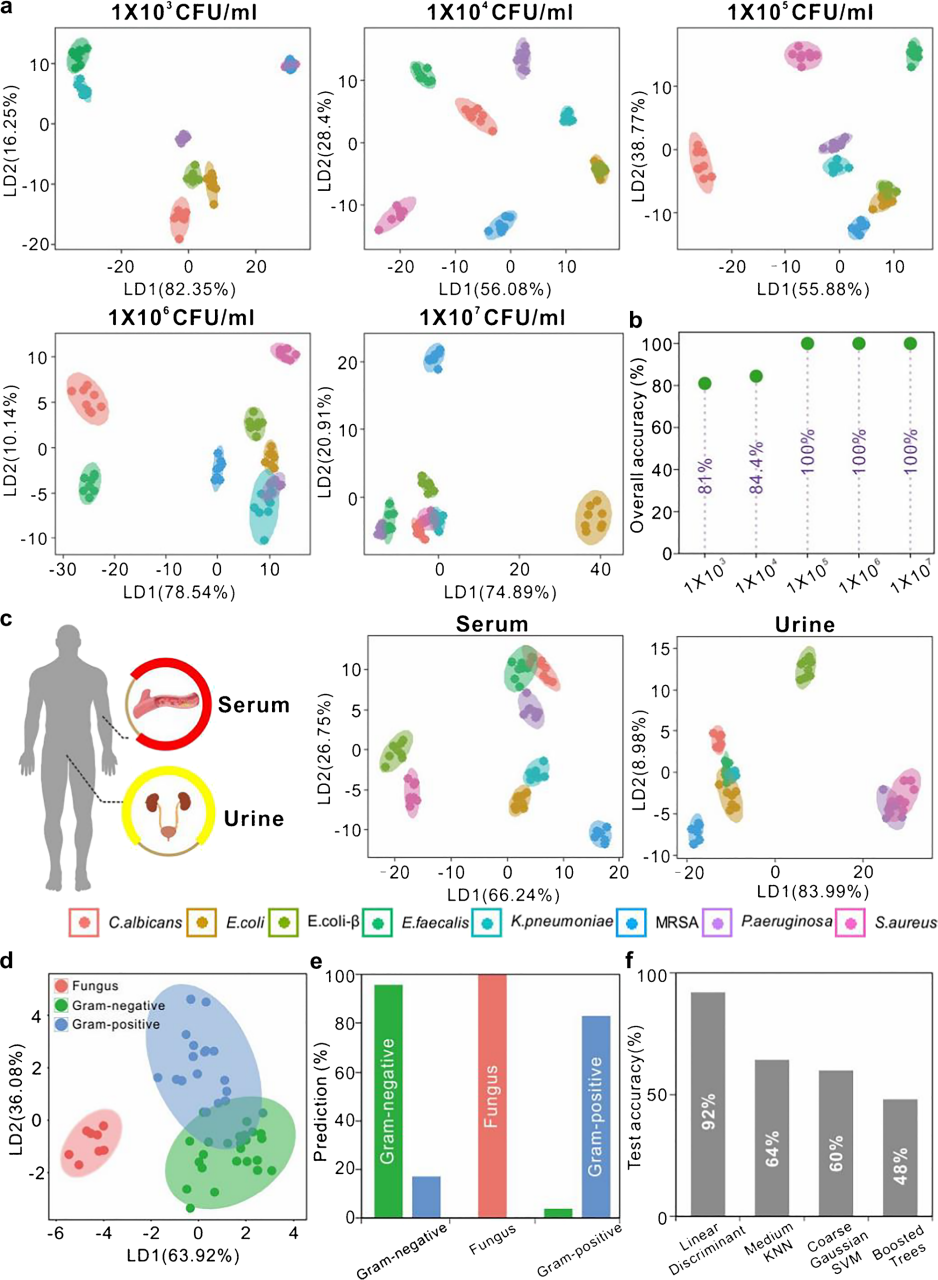
Discrimination of multiple pathogenic microorganisms in body fluids and determination of the Gram-status of the pathogenic microorganisms using the custom cross-response sensing array. (**a**) Different concentrations of pathogenic microorganism were detected, such as 1 × 10^3^ CFU/mL, 1 × 10^4^ CFU/mL, 1 × 10^5^ CFU/mL, 1 × 10^6^ CFU/mL, 1 × 10^7^ CFU/mL, respectively. (**b**) The relationship between microorganism concentration and overall accuracy. (**c**) Simultaneous discrimination for multiple pathogenic microorganisms in the different body fluids. The test body fluids contain serum and urine. The concentration of pathogenic microorganism was 1 × 10^6^ CFU/mL. Measure the classification performance (**d**) and the accuracy (**e**) using the linear discriminant analysis. (**f**) The performance of the proposed method is compared to different machine learning algorithms

To demonstrate the sensitivity of our approach, we present the discrimination capacity of multiple pathogenic microorganisms at 1 × 10^3^ CFU/mL, 1 × 10^4^ CFU/mL, 1 × 10^5^ CFU/mL, 1 × 10^6^ CFU/mL, and 1 × 10^7^ CFU/mL, respectively. The result shows that the approach can clearly distinguish different pathogenic microorganisms, as shown in Fig. 4a; the ellipsoids in Fig. 4a represent the confidence interval at 95%. The recognition accuracy achieves 81% at 1 × 10^3^ CFU/mL of pathogenic microorganisms, as shown in Fig. 4b. Thus, our cross-response sensing array is extremely sensitive. Moreover, we individually probe their detection range for each microorganism. We find that LD2 (the second linear discrimination factor) is not exceeding 40%, and LD1 (the first linear discriminant factor) can be simply used to quantify the concentrations of microorganisms [30], as shown in Figures S5 and S6. For each microorganism, the detection concentration is 10^5^ ~ 10^8^ CFU/mL for *E. coli*, 10^2^ ~ 10^7^ CFU/mL for *E. coli-β*, 10^3^ ~ 10^8^ CFU/mL for *S. aureus*, 10^3^ ~ 10^7^ CFU/mL for MRSA, 10^2^ ~ 10^8^ CFU/mL for *P. aeruginosa*, 10^3^ ~ 10^8^ CFU/mL for *E. faecalis*, 10^2^ ~ 10^8^ CFU/mL for *K. pneumoniae*, and 10^3^ ~ 10^8^ CFU/mL for *C. albicans*. Several methods have been developed to detect multiple microorganism, including Colorimetric (UV–vis) [44, 45], SERS [46, 47], Electrochemical [48–51], and Fluorescence spectroscopy [52, 53]. The detection throughput and range of this method are significant advantages to reported methods for simultaneous detection (Table S3). What is more, the detection range of this method meets the standard requirements of bacterium for antimicrobial susceptibility test (AST), which is around 1.5 × 10^8^ CFU/mL based on World Health Organization (WHO) standard [54]. This sensing array is further challenged by various media that are critical to their practical application. As a proof of concept, we tested the microbes in the serum and urine. We can obtain a set of the fluorescent incremental of each microbial fluorescence response through the equation of Δ*I* = *I* − *I*_0_ for each body fluid, as shown in Figures S8 and S9. This sensing array showed excellent performance in the different body fluids, such as serum and urine, as shown in Fig. 4c and Tables S4 to S6; the ellipsoids in Fig. 4c represent the confidence interval at 95%. As shown in Tables S7 to S8, the apparent recovery of microorganism in the different solutions for each sample is over the range of 90.47–115.56%. These results suggest that our sensing array has the potential to be applied to body fluid samples.

A critical challenge in pathogenic microorganisms’ therapy is the accurate determination of pathogenic bacteria’s Gram-status, which determines initial medication regimens [43, 55]. Therefore, we divide eight different pathogenic microorganisms into three groups, which are Gram-negative bacteria, Gram-positive bacteria, and fungus, to test our cross-response sensing array in this application. The different types of Gram-status of pathogenic microorganisms also show differential fluorescence responsive differential profiling (fluorescent incremental Δ*I* normalized), as shown in Figures S7a to c. *E. coli*, *E. coli*-β, *P. aeruginosa*, and *K. pneumoniae* are Gram-negative bacteria. *S. aureus*, MRSA, and *E. faecalis* are Gram-positive bacteria. *C. albicans* are fungus. The molecular response differential profiling approach’s performance is shown in Fig. 4d to f. This finalized training matrix with 384 data points from 64 test cases (6 sensing elements × 8 microorganisms × 8 replicates) produces linear discrimination factors of 63.92 and 36.08, and the overall recognition accuracy is 92% in LDA (Fig. 4d and f); the ellipsoids in Fig. 4d represent the confidence interval at 95%. The multi-class identification performance of the LDA for the three Gram-status is shown in Fig. 4e. The machine learning approach based on a cross-response sensing array identifies Gram-status with high specificity. Furthermore, different machine learning algorithms’ performances are compared (Fig. 4f): LDA, medium KNN, coarse Gaussian SVM, and boosted trees. The result shows that the LDA algorithm outperforms the other algorithms in the accuracy with a substantial degree.

The advantage of array-based sensors over traditional single-component sensors is that they can spontaneously distinguish a variety of targets, allowing to break the traditional limitation of “lock and key” principle and making it possible to simultaneously detect multi-targets. Although sensing array offers a huge opportunity for the development of bacterial detection sensors, the current fingerprint pattern–recognition sensing approach still needs to be further improved to fully meet the requirements of practical applications. First, small changes in molecular receptors or interfering species can lead to huge deviations in the final output. To solve this problem, more efforts should be put on developing more effective and selective receptors, which will hopefully reduce interference and improve specificity. Second, delving into the contribution of each component in a multi-component sensor array optimizes the performance of the component of the sensing array. The number of components is directly related to the effort, time-consuming process, and accuracy of identifying the results. Third, the current biosensor array can only identify bacteria in existing databases and is not suitable for unknown and untested microorganisms. The refinement and expansion of the database and the development of intermediate laws for the prediction of bacterial species are needed. In addition, a lot of effort is required to realize real-world applications of array sensing. Current research mainly relies on pure bacteria rather than the original sample for bacterial identification, which might be caused by the low microbial concentration and large interference of the original sample. Although many bacterial sensors have been proven in laboratory studies, it is still far from practical application due to their time-consuming procedures and instrument-based readouts. Future research should focus on refinements to simplify procedures and readouts, enabling timeliness, low cost, and convenient visualization of biosensor arrays.

## Conclusion

In summary, we have developed a molecular response differential profiling based on a custom cross-response sensing array for rapid and accurate pathogenic microbial taxonomic identification. The custom cross-response sensing array’s sensing elements consist of different fluorescence-labeled ssDNA molecular and different two-dimensional nanomaterial (2D-n) fluorescence quenchers. In this work, we confirm that the molecular response differential profiling for different microorganisms is derived from the competitive response capacity of varying sensing elements in the sensing array to various pathogenic microorganisms, including drug resistance microorganisms, which proves the ability of our approach to directly recognize and phenotype pathogenic microorganism. This molecular response differential profiling based on a custom sensing array has several inherent advantages. First, the sensing array is diverse and customizable, ensuring extract information differentiation and overcoming dependence on big data. Second, the sensing element’s silhouette coefficient visualization shows each sensing element to the degree of influence on the classification results, ensuring the approach’s identification process is visible. Third, the approach shows good practicability, such as accurately determining the phenotyping and Gram-state of pathogenic bacteria. Overall, the cross-response sensing array converts the target analyte into the unique molecular response differential profiling, as a new way of developing biomedical sensing arrays. Therefore, this study provides a highly generic idea and tool for precision medicine application.

## Supplementary Information

Below is the link to the electronic supplementary material.

Supplementary file1 (DOCX 2205 KB)
