# Supplementary material for: 2D nanomaterial sensing array using machine learning for differential profiling of pathogenic microbial taxonomic identification
Source: Mikrochim Acta. 2022 Jul 6;189(8):273. doi: 10.1007/s00604-022-05368-5 (PMC9259531; doi:10.1007/s00604-022-05368-5)
Supplement: Supplementary file 1 — Supplementary file1 (DOCX 2205 KB) [file 604_2022_5368_MOESM1_ESM.docx]

Supporting Information

**2D Nanomaterial Sensing Array using Machine Learning for Differential Profiling of Pathogenic Microbial Taxonomic Identification**

Zhijun Li,^[a]+^ Yizhou Jiang,^[a]+^ Shihuan Tang,^[b]+^ Haixia Zou,^[a]^ Wentao Wang,^[a]^ Guangpei Qi,^[a]^ Hongbo Zhang,^[c]*^ Kun Jin,^[a]^ Yuhe Wang,^[d]^ Hong Chen,^[e]^ Liyuan Zhang,^[d][f]*^ Xiangmeng Qu^[a]*^

1. Key Laboratory of Sensing Technology and Biomedical Instruments of Guangdong Province, School of Biomedical Engineering, Sun Yat-Sen University, Shenzhen 518017, China
2. Department of Clinical Laboratory, The Seventh Affiliated Hospital of Sun Yat-Sen University, Shenzhen 518017, Guangdong, China

[c] Pharmaceutical Sciences Laboratory, Åbo Akademi University; Turku Bioscience Centre, University of Turku and Åbo Akademi University, Turku, 20520 Finland

[d] School of Petroleum Engineering, State Key Laboratory of Heavy Oil Processing, China University of Petroleum (East China), Qingdao 266580, China

[e] Pen-Tung Sah Institute of Micro-Nano Science and Technology, Xiamen University, Xiamen 361005, China

[f] Harvard John A. Paulson School of Engineering and Applied Sciences, Harvard University, Cambridge, MA 02138, USA

[+] These authors contributed equally to this work.

Corresponding author: E-mail: [quxm5@mail.sysu.edu.cn](mailto:quxm5@mail.sysu.edu.cn) ; [Hongbo.zhang@abo.fi](mailto:Hongbo.zhang@abo.fi) ; [liyuanzhang@seas.harvard.edu](mailto:liyuanzhang@seas.harvard.edu)

**Linear Discriminant Analysis.**

**LDA R package to visualize the classification.**

library(devtools)

library(ggord)

library(MASS)

library(ggplot2)

data<-read.csv ("Bacteria.csv",sep=",",header=T)

data$group <- as.factor(data$group)

model1=lda(group~.,data=data)

model1

plot (model1, dimen=1)

ld<-predict(model1) $x

ord <- lda (group ~., data)

p <- ggord (ord, data$group,arrow=0, vec_ext =0, size =5,txt=NULL, poly = FALSE, coord_fix = F)

p + theme (panel.grid = element_blank()) [1]

Linear discriminant analysis (LDA) can distinguish different objects or assign new objects to appropriate classes, successfully applied to separate categories of objects based on responses from sensor arrays [2-5]. Here, the input variables are the responses of our sensors. The role of the LDA is to obtain optimal discriminants that maximize the distance between the classes and minimize the distance within the categories [6]. The measure of types variation can be calculated as

$$S_{k}=\frac{1}{n_{k}-1}\sum_{i=1}^{n_{k}} [(\bar{x}_{k_{i}}-\bar{x}_{k}){(\bar{x}_{k_{i}}-\bar{x}_{k})}^{T}]$$

$$S_{p}=\frac{\sum_{k=1}^{m} [(n_{k}-1)S_{k}]}{\sum_{k=1}^{m} n_{k}-m}$$

$$W=[\sum_{k=1}^{m} n_{k}-m]S_{p}$$

Furthermore, the measure of variation between classes can be obtained as

$$\bar{x}=\frac{\sum_{k=1}^{m} n_{k}\bar{x}_{k}}{\sum_{k=1}^{m} n_{k}}$$

$$B=\sum_{k=1}^{m} (\bar{x}_{k}-\bar{x}){(\bar{x}_{k}-\bar{x})}^{T}$$

where $n_{k}$ is the total sample size of the k-th class, $\bar{x}_{k_{i}}$ denotes the average value of the i-th column of all samples of the k-th class, $\bar{x}_{k}$ and $\bar{x}$ means the average value of all samples of the k-th class and the average value of all samples, respectively.

By calculating the eigenvectors of W^-1^B, we can obtain the coefficients of the discriminants. When using six sensing elements (A20-GO, T20-GO, C20-GO, A20-WS_2_, T20-WS_2_, and C20-WS_2_) to identify different microorganisms, the linear discriminants are as follows:

$$LD1=3.197506\times I_{A20-GO}+5.658523\times I_{T20-GO}+76.916670\times I_{C20-GO}+12.476496\times I_{A20-WS2}-7.669155\times I_{T20-WS2}-12.708010\times I_{C20-WS2}-21.6216015$$

$$LD2=0.9755376\times I_{A20-GO}-13.2933252\times I_{T20-GO}+34.1305541\times I_{C20-GO}-24.7344150\times I_{A20-WS2}-8.8753390\times I_{T20-WS2}-3.6740100\times I_{C20-WS2}+13.190898$$

And for determining the Gram-status of the pathogenic microorganisms, the linear discriminants are as follows:

$$LD1=-5.806828\times I_{A20-GO}-13.467988\times I_{T20-GO}+6.795376\times I_{C20-GO}-9.445977\times I_{A20-WS2}+16.712232\times I_{T20-WS2}+19.441186\times I_{C20-WS2}-4.883628$$

$$LD2=6.52588538\times I_{A20-GO}-28.21000200\times I_{T20-GO}+10.166037533\times I_{C20-GO}+14.75731373\times I_{A20-WS2}+0.03077778\times I_{T20-WS2}-8.24180326\times I_{C20-WS2}+0.49886164$$

**Silhouette Coefficient.**

Silhouette Coefficient (SC) is a way to evaluate the effectiveness of clustering. The extent of cohesion and separation (separate from other characteristic variables) of the probe is considered by SC. SC represents different classification abilities that six probes loaded one by one.

The calculation formula of the Silhouette coefficient is as follow:

$$SC=\frac{1}{N}\sum_{i=1}^{N} \frac{b\left( i \right)-a(i)}{\max\left\{ a\left( i \right),b\left( i \right) \right\}}$$

In which a(*i*) = Average $\left\{ The distance of the Vector i to all the other points in the cluster that i belongs to \right\};$

b(*i*) = Min $\left\{ The average distance of the Vector i to all the points in the cluster nearest to i \right\}$.

We can draw a conclusion from the formula that the value of SC is between [-1, 1]. The closer the SC is to 1, the better the cohesion and separation levels of the probe is.

**
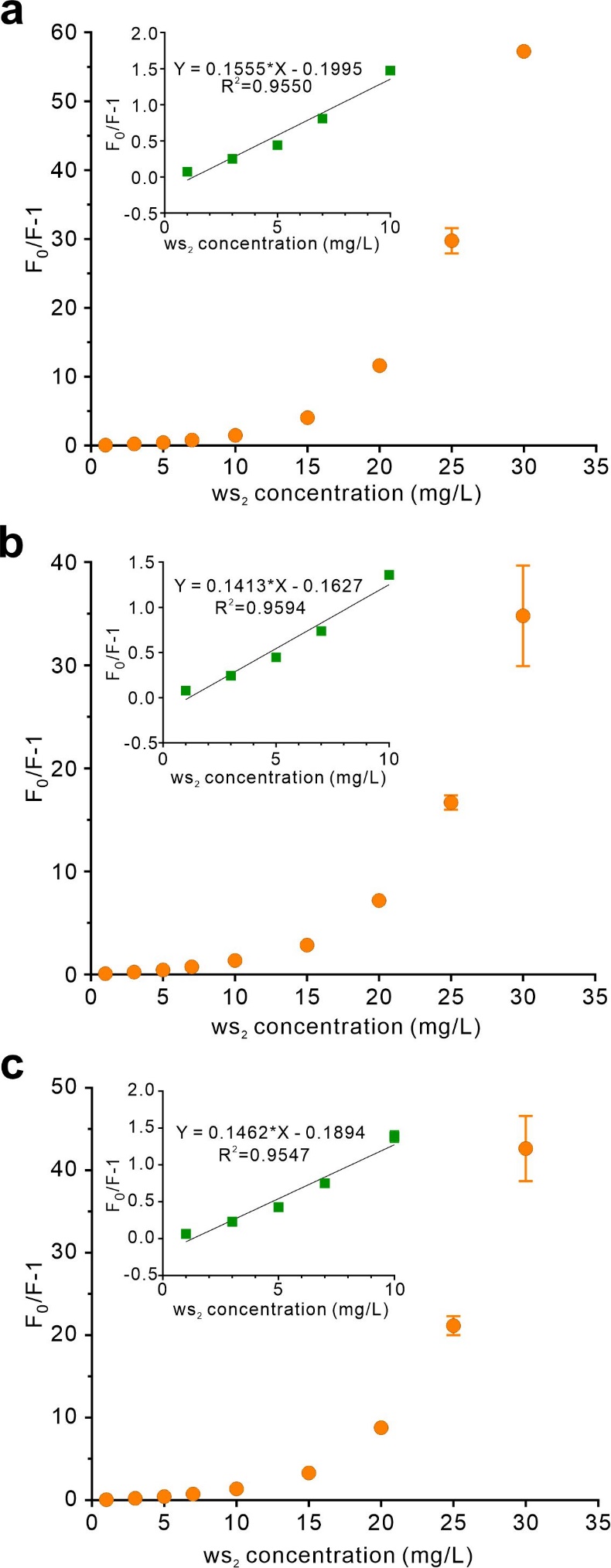
**

**Figure S1.** Stern-Volmer under different WS_2_ concentration; a) FAM-T20; b) FAM-A20; c) FAM-C20.


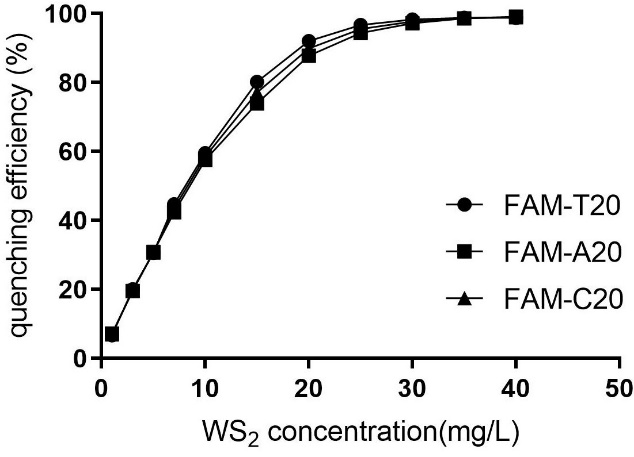


**Figure S2**. The quench efficiency of WS_2_ with different concentration to different kind of FAM-DNA.


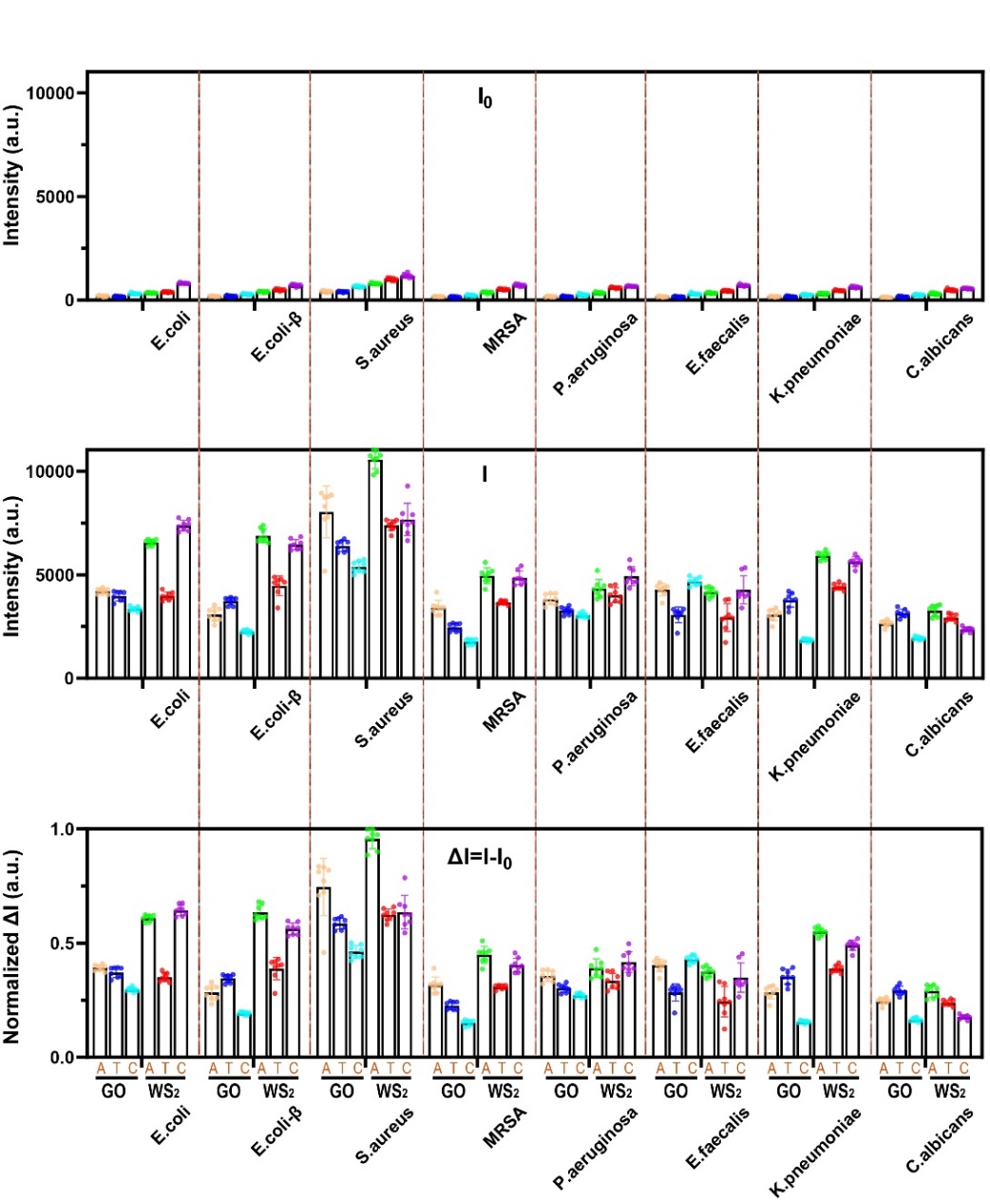


**Figure S3**. A set of the fluorescent intensity of the initial (I_0_(a.u.)), the relative (I(a.u.)) and the normalized relative increment (ΔI) response is measured with a microplate reader in the absence or presence of microorganism, respectively.

**
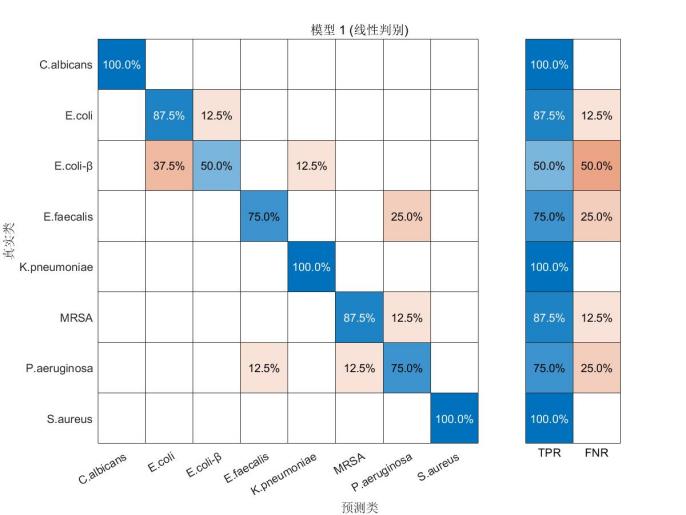

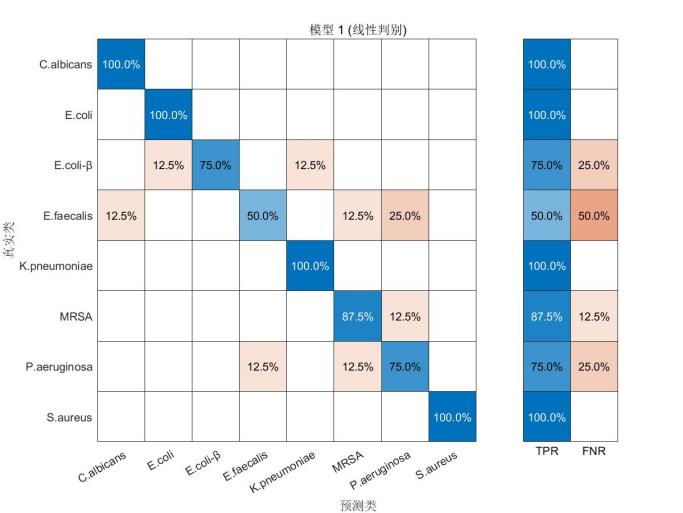
**

1. **(b)**

**
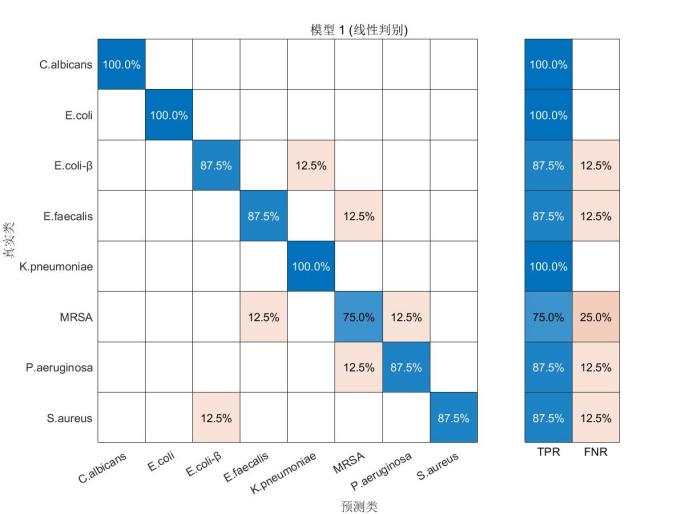

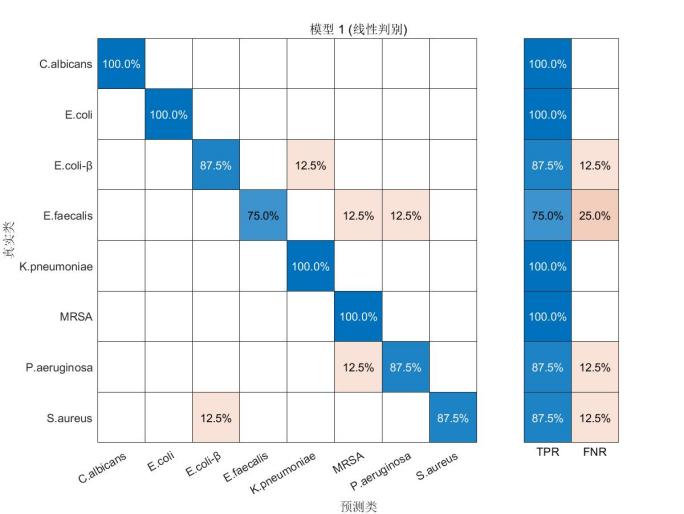
**

**(c) (d)**

**
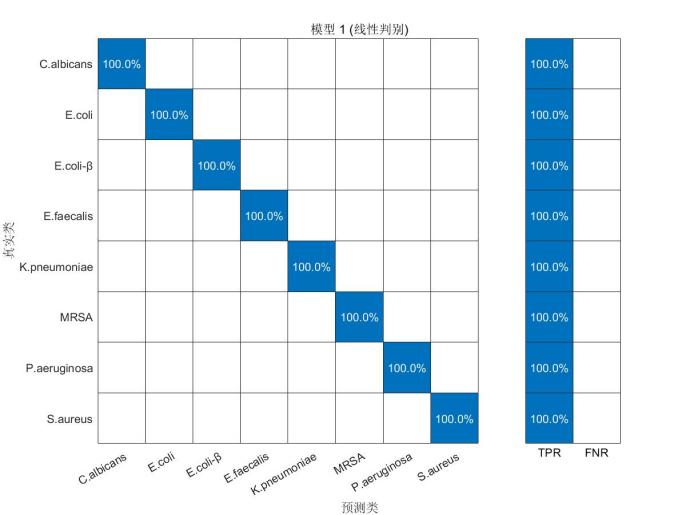
**

**(e)**

**Figure S4.** Confusion matrix of increased the number of probes for the discrimination of eight bacteria. (a) Two types of sensing elements: T20-WS_2_ and A20-WS_2_, (b) three sensing elements: T20-WS_2_, A20-WS_2_, and C20-WS_2_, (c) four sensing elements: A20-GO, T20-WS_2_, A20-WS_2_, and C20-WS_2_, (d) five sensing elements: T20-GO, A20-GO, T20-WS_2_, A20-WS_2_, and C20-WS_2_, (e) six sensing elements: T20-GO, A20-GO, C20-GO, T20-WS_2_, A20-WS_2_, C20-WS_2_.


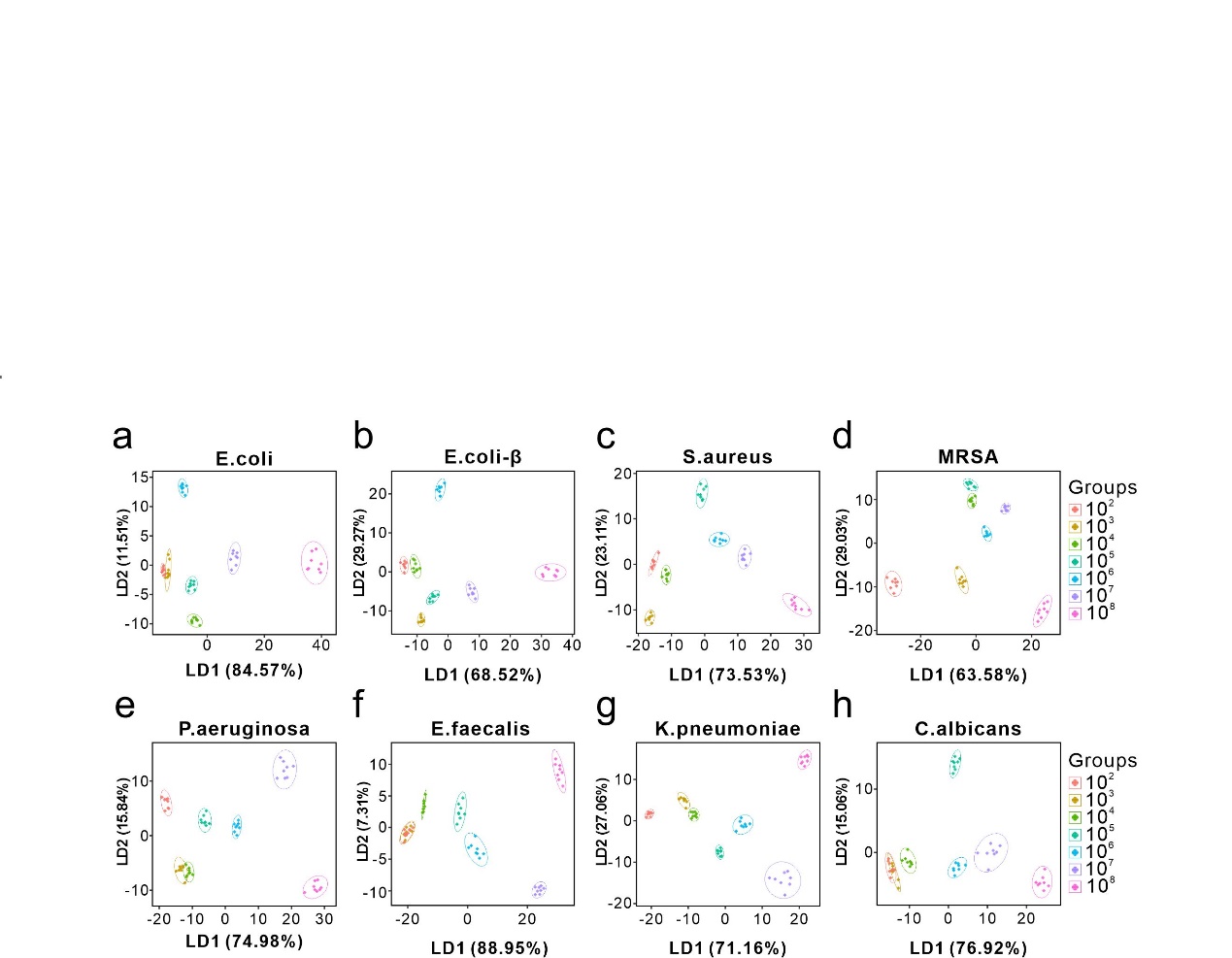


**Figure S5.** Discrimination of various densities of each microorganism.


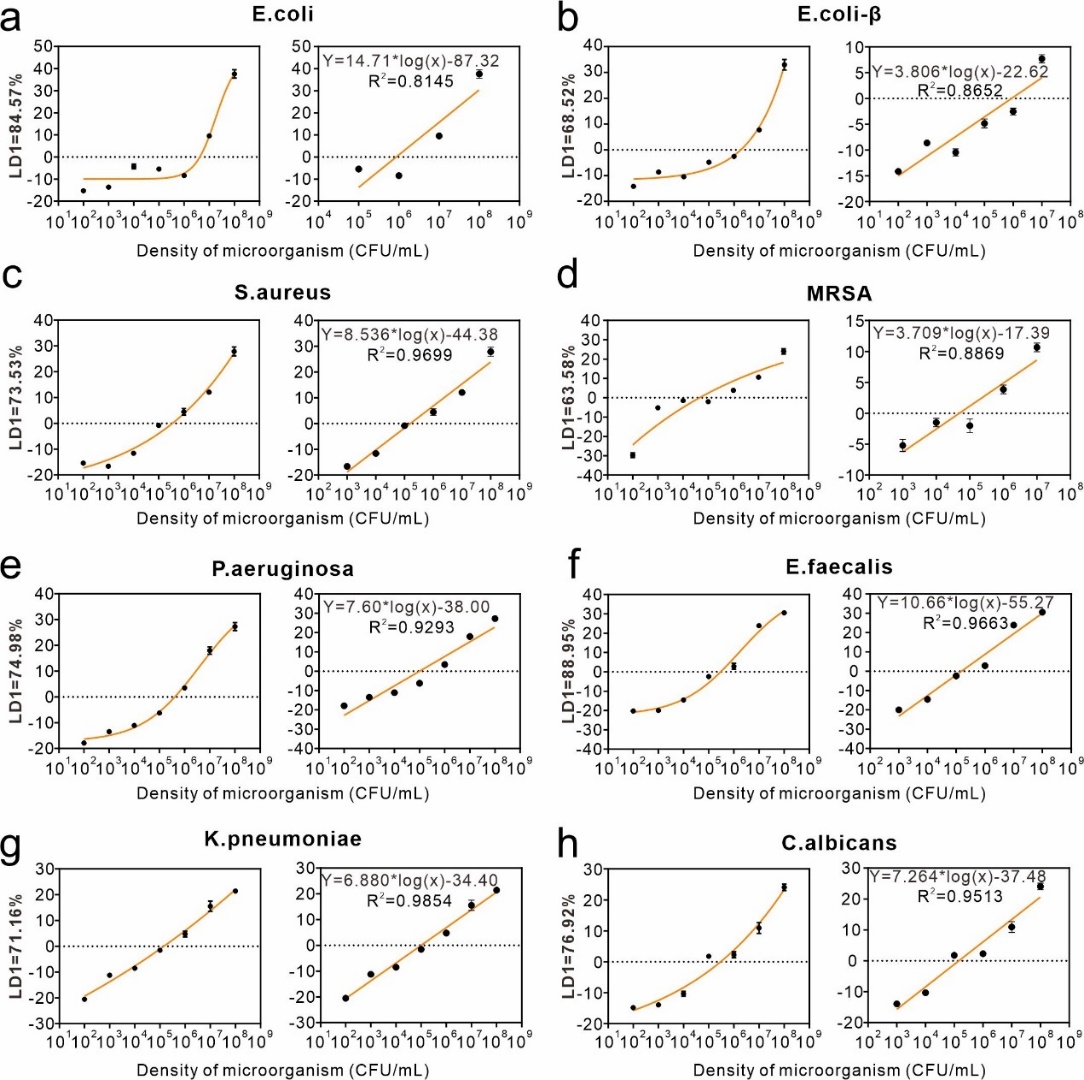


**Figure S6.** Detection range of 8 microorganism using sensing array.


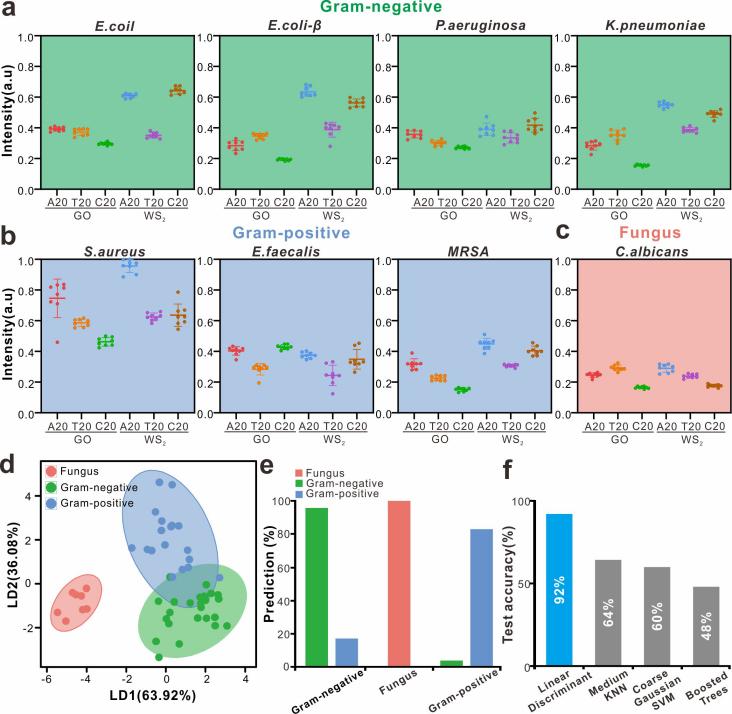


**Figure S7.** Response profiling of Gram-negative (a), Gram-positive (b), and Fungus (c).


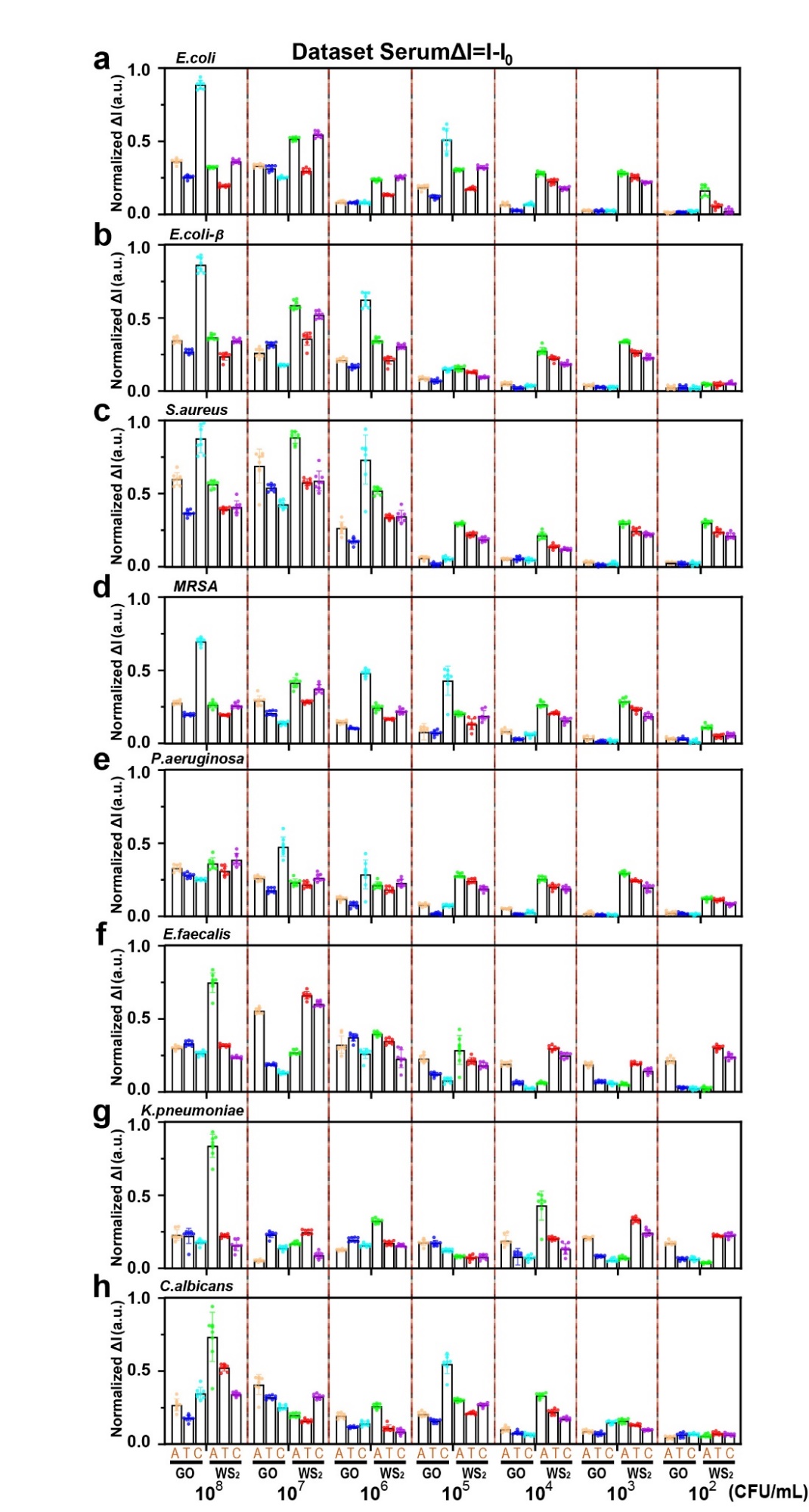


**Figure S8**. The fluorescent normalized relative increment (ΔI) response by calculation (ΔI=I-I_0_) for each microorganism in serum.

**
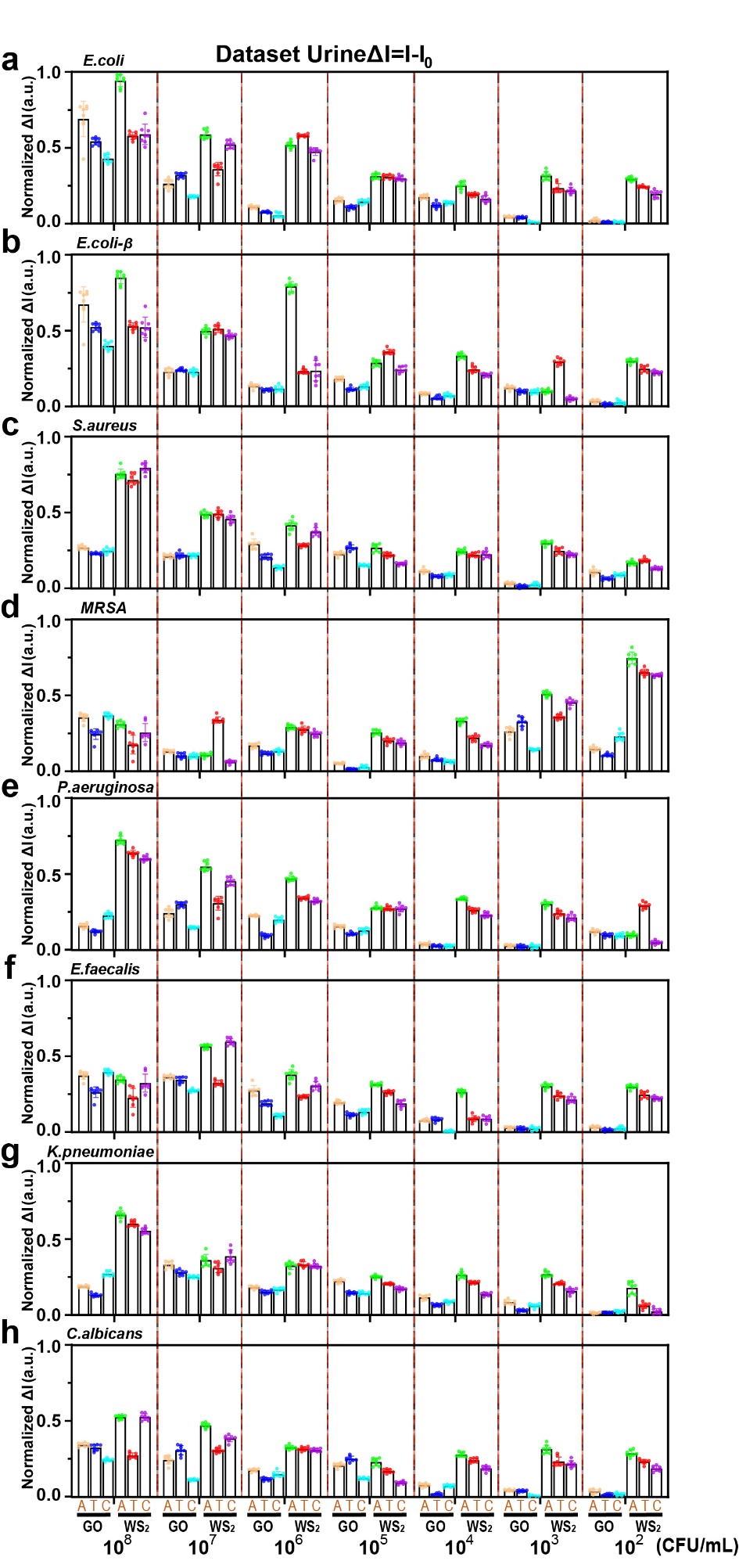
**

**Figure S9**. The fluorescent normalized relative increment (ΔI) response by calculation (ΔI=I-I_0_) for each microorganism in urine.

**Table S1. DNA sequences used in this work.**

| oligonucleotide sequences | 5’to 3’ |
| --- | --- |
| A20 | 6-FAM/AAAAA AAAAA AAAAA AAAAA |
| T20 | 6-FAM/ TTTTT TTTTT TTTTT TTTTT |
| C20 | 6-FAM/ CCCCC CCCCC CCCCC CCCCC |

**Table S2. The microorganisms used in this work.**

| specific names | specific numbers |
| --- | --- |
| Methicillin-resistant Staphylococcus aureus, MRSA | ATCC43300 |
| *Staphylococcus aureus, S.aureus* | ATCC6538 |
| *Enterococcus faecalis, E.faecalis* | ATCC29212 |
| *Escherichia coli, E.coli* | ATCC25922 |
| *β-lactams-resistant E.coli，E.coli-β* | ATCC35218 |
| *Klebsiella pneumoniae, K.pneumoniae* | CMCC(B)46117 |
| *Pseudomonas aeruginosa, P.aeruginosa* | ATCC27853 |
| *Candida albicans, C.albicans* | ATCC10231 |

**Table S3. Analytical performances of recent methods for simultaneous detection of multiple microorganism.**

| Detection method | nanomaterials | Detection range  (CFU/mL) | LOD  (CFU/mL) | Species | | Sensing array | Ref |
| --- | --- | --- | --- | --- | --- | --- | --- |
| Colorimetric  (UV–vis) | AgNPs | 5.0×10^4^-1.0×10^7^ | 0.9×10^4^ | *E. coli* | Yes | | [7] |
|  | AuNPs,  AgNPs | 1.0×10^2^-1.0×10^8^ | 1.0×10^2^ | *S. aureus,*  *MRSA,*  *Listeria,*  *S. agalactiae,*  *E. faecalis,*  *E. coli,*  *Klebsiella,*  *Proteus,*  *E. aerogenes,*  *P. aeruginosa* | Yes | | [8] |
| SERS-based assay | 4-MPBA functionalized AgNPs | 500-2000 | / | *E. coli,*  *S. aureus* | No | | [9] |
|  | AgNPs@4-mercaptobenzonitrile@MPNs tags | 1.0×10^2^-1.0×10^8^ | 1.0×10^2^ | *E. coli* O157:H7*,*  *S. aureus* | No | | [10] |
| Electrochemical | Au microelectrode | / | 400 | *E. coli* | No | | [11] |
|  | Magnetic  nanoparticles-  antibody  conjugates | 7.4× 10^4^-7.4×10^7^ | 7.4×10^4^ | *E. coli* | Yes | | [12] |
|  | magnetic beads (MBs) a screen-printed interdigitated microelectrode (SP-IDME) | 1.0×10^2^-1.0×10^6^ | 2.05 × 10^3^  1.04 × 10^3^ | *E. coli*  *S. Typhimurium* | No | | [13] |
|  | Au@PtNPs-laden neutral red functionalized reduced graphene oxide | 8.9×10^3^-8.9×10^9^ | 2.84×10^3^ | *E. coli* | No | | [14] |
| Fluorescence spectroscopy | Vancomycin functionalized gold nanoclusters and aptamer-modified gold nanoparticles as the energy donor and acceptor | 20-1.0×10^8^ | 10 | *S. aureus* in artificial complex specimen | No | | [15] |
|  | FPs/AuNP@Fe | / | 1.0×10^6^ | *Listeria monocytogenes,*  *Proteus mirabilis,*  *Enterococcus faecalis,*  *Klebsiella pneumonia ,*  *Staphylococcus. aureus ,*  *Pseudomonas aeruginosa ,*  *E coli,*  *Escherichia typhi,*  *Shigella dysenteriae* | Yes | | [16] |
|  | 2-dimensional nanomaterial (2D-n) with fluorescent labeled DNA | 1.0×10^5^-1.0×10^8^ | 1.0×10^5^ | *E.coli* | Yes | | This work |
|  |  | 1.0×10^2^-1.0×10^7^ | 1.0×10^2^ | *E.coli-β* |  |  |  |
|  |  | 1.0×10^3^-1.0×10^8^ | 1.0×10^3^ | *S.aureus* |  |  |  |
|  |  | 1.0×10^3^-1.0×10^7^ | 1.0×10^3^ | *MRSA* |  |  |  |
|  |  | 1.0×10^2^-1.0×10^8^ | 1.0×10^2^ | *P.aeruginosa* |  |  |  |
|  |  | 1.0×10^3^-1.0×10^8^ | 1.0×10^3^ | *E.faecalis* |  |  |  |
|  |  | 1.0×10^2^-1.0×10^8^ | 1.0×10^2^ | *K.pneumoniae* |  |  |  |
|  |  | 1.0×10^3^-1.0×10^8^ | 1.0×10^3^ | *C.albicans* |  |  |  |

**Table S4. Classification performance of different sensing arrays in PBS.**

| Sensor elements | Precision | Recall | F1 score |
| --- | --- | --- | --- |
| T20-WS_2_, A20-WS_2_ | 0.85 | 0.84 | 0.84 |
| T20-WS_2_, A20-WS_2_, C20-WS_2_ | 0.86 | 0.86 | 0.86 |
| A20-GO, T20-WS_2_, A20-WS_2_, C20-WS_2_ | 0.91 | 0.91 | 0.91 |
| T20-GO, A20-GO, T20-WS_2_, A20-WS_2_, C20-WS_2_ | 0.93 | 0.92 | 0.92 |
| T20-GO, A20-GO, C20-GO, T20-WS_2_, A20-WS_2_, C20-WS_2_ | 1 | 1 | 1 |

**Table S5. Classification performance of different sensing arrays in serum.**

| Sensor elements | Precision | Recall | F1 score |
| --- | --- | --- | --- |
| T20-WS_2_, A20-WS_2_ | 0.5 | 0.5 | 0.5 |
| T20-WS_2_, A20-WS_2_, C20-WS_2_ | 0.64 | 0.63 | 0.63 |
| A20-GO, T20-WS_2_, A20-WS_2_, C20-WS_2_ | 0.92 | 0.91 | 0.91 |
| T20-GO, A20-GO, T20-WS_2_, A20-WS_2_, C20-WS_2_ | 0.95 | 0.94 | 0.95 |
| T20-GO, A20-GO, C20-GO, T20-WS_2_, A20-WS_2_, C20-WS_2_ | 1 | 1 | 1 |

**Table S6. Classification performance of different sensing arrays in urine.**

| Sensor elements | Precision | Recall | F1 score |
| --- | --- | --- | --- |
| T20-WS_2_, A20-WS_2_ | 0.77 | 0.77 | 0.77 |
| T20-WS_2_, A20-WS_2_, C20-WS_2_ | 0.84 | 0.84 | 0.84 |
| A20-GO, T20-WS_2_, A20-WS_2_, C20-WS_2_ | 0.89 | 0.88 | 0.88 |
| T20-GO, A20-GO, T20-WS_2_, A20-WS_2_, C20-WS_2_ | 0.93 | 0.92 | 0.92 |
| T20-GO, A20-GO, C20-GO, T20-WS_2_, A20-WS_2_, C20-WS_2_ | 0.97 | 0.95 | 0.96 |

**Table S7. Apparent recovery experiments of microorganism in serum**

| Species | Add amount（CFU/mL） | Detected（CFU/mL） | Recoveries (%) |
| --- | --- | --- | --- |
| *E.coli* | 10^5^ | 96463.80 | 96.46 |
| *E.coli* | 10^6^ | 904674.15 | 90.47 |
| *E.coli* | 10^7^ | 9032591.56 | 90.32 |
| *E.coli* | 10^8^ | 94520683.29 | 94.52 |
| *E.coli-β* | 10^2^ | 106.62 | 106.62 |
| *E.coli-β* | 10^3^ | 994.57 | 99.46 |
| *E.coli-β* | 10^4^ | 9390.20 | 93.90 |
| *E.coli-β* | 10^5^ | 105595.86 | 105.60 |
| *E.coli-β* | 10^6^ | 946434.11 | 94.64 |
| *E.coli-β* | 10^7^ | 9867784.42 | 98.68 |
| *S.aureus* | 10^3^ | 996.06 | 96.61 |
| *S.aureus* | 10^4^ | 10657.31 | 106.57 |
| *S.aureus* | 10^5^ | 92226.29 | 92.23 |
| *S.aureus* | 10^6^ | 1045232.12 | 104.52 |
| *S.aureus* | 10^7^ | 9418733.36 | 94.187 |
| *S.aureus* | 10^8^ | 102512749.50 | 102.51 |
| *MRSA* | 10^3^ | 1103.06 | 110.3 |
| *MRSA* | 10^4^ | 10340.92 | 104.41 |
| *MRSA* | 10^5^ | 90825.91 | 90.83 |
| *MRSA* | 10^6^ | 1157787.91 | 115.78 |
| *MRSA* | 10^7^ | 10753377.47 | 107.53 |
| *P.aeruginosa* | 10^2^ | 113.29 | 113.29 |
| *P.aeruginosa* | 10^3^ | 977.53 | 97.75 |
| *P.aeruginosa* | 10^4^ | 9939.59 | 99.40 |
| *P.aeruginosa* | 10^5^ | 106246.78 | 106.25 |
| *P.aeruginosa* | 10^6^ | 929867.47 | 92.99 |
| *P.aeruginosa* | 10^7^ | 9412049.67 | 94.12 |
| *P.aeruginosa* | 10^8^ | 106246783.09 | 106.25 |
| *E.faecalis* | 10^3^ | 1113.09 | 111.31 |
| *E.faecalis* | 10^4^ | 10973.31 | 109.73 |
| *E.faecalis* | 10^5^ | 99354.09 | 99.35 |
| *E.faecalis* | 10^6^ | 1069253.49 | 106.93 |
| *E.faecalis* | 10^7^ | 11507357.61 | 115.07 |
| *E.faecalis* | 10^8^ | 99784230.76 | 99.78 |
| *K.pneumoniae* | 10^2^ | 93.05 | 93.06 |
| *K.pneumoniae* | 10^3^ | 1058.54 | 105.85 |
| *K.pneumoniae* | 10^4^ | 9606.34 | 96.06 |
| *K.pneumoniae* | 10^5^ | 103750.06 | 103.75 |
| *K.pneumoniae* | 10^6^ | 1040978.74 | 104.10 |
| *K.pneumoniae* | 10^7^ | 10836367.40 | 108.36 |
| *K.pneumoniae* | 10^8^ | 112804280.91 | 112.80 |
| *C.albicans* | 10^3^ | 1057.38 | 105.73 |
| *C.albicans* | 10^4^ | 9162.29 | 91.62 |
| *C.albicans* | 10^5^ | 105202.59 | 105.20 |
| *C.albicans* | 10^6^ | 967570.95 | 96.76 |
| *C.albicans* | 10^7^ | 9786756.13 | 97.88 |
| *C.albicans* | 10^8^ | 112372694.07 | 112.37 |

**Table S8. Apparent recovery experiments of microorganism in urine**

| Species | Add amount（CFU/mL） | Detected（CFU/mL） | Recoveries(%) |
| --- | --- | --- | --- |
| *E.coli* | 10^5^ | 91321.08 | 91.32 |
| *E.coli* | 10^6^ | 964638.03 | 96.46 |
| *E.coli* | 10^7^ | 9737405.05 | 97.37 |
| *E.coli* | 10^8^ | 105962685.68 | 105.96 |
| *E.coli-β* | 10^2^ | 93.45 | 93.45 |
| *E.coli-β* | 10^3^ | 1035.71 | 103.57 |
| *E.coli-β* | 10^4^ | 9504.51 | 95.05 |
| *E.coli-β* | 10^5^ | 98797.31 | 98.80 |
| *E.coli-β* | 10^6^ | 1033208.87 | 103.32 |
| *E.coli-β* | 10^7^ | 10546816.44 | 105.47 |
| *S.aureus* | 10^3^ | 932.77 | 93.28 |
| *S.aureus* | 10^4^ | 10179.63 | 101.80 |
| *S.aureus* | 10^5^ | 92725.19 | 92.73 |
| *S.aureus* | 10^6^ | 1082536.03 | 108.25 |
| *S.aureus* | 10^7^ | 9650194.85 | 96.50 |
| *S.aureus* | 10^8^ | 109074338.22 | 109.07 |
| *MRSA* | 10^3^ | 1155.63 | 115.56 |
| *MRSA* | 10^4^ | 10405.32 | 104.05 |
| *MRSA* | 10^5^ | 107400.34 | 107.40 |
| *MRSA* | 10^6^ | 1074670.37 | 107.47 |
| *MRSA* | 10^7^ | 9322525.22 | 93.23 |
| *P.aeruginosa* | 10^2^ | 110.18 | 110.18 |
| *P.aeruginosa* | 10^3^ | 977.53 | 97.75 |
| *P.aeruginosa* | 10^4^ | 9939.59 | 99.40 |
| *P.aeruginosa* | 10^5^ | 106569.17 | 106.57 |
| *P.aeruginosa* | 10^6^ | 955571.43 | 95.56 |
| *P.aeruginosa* | 10^7^ | 10592537.25 | 105.93 |
| *P.aeruginosa* | 10^8^ | 98198592.54 | 98.20 |
| *E.faecalis* | 10^3^ | 1076.20 | 107.62 |
| *E.faecalis* | 10^4^ | 10646.44 | 106.46 |
| *E.faecalis* | 10^5^ | 95565.29 | 95.57 |
| *E.faecalis* | 10^6^ | 955571.43 | 95.56 |
| *E.faecalis* | 10^7^ | 10351645.26 | 103.52 |
| *E.faecalis* | 10^8^ | 110685327.98 | 110.69 |
| *K.pneumoniae* | 10^2^ | 103.06 | 103.06 |
| *K.pneumoniae* | 10^3^ | 1044.47 | 104.45 |
| *K.pneumoniae* | 10^4^ | 9014.50 | 90.15 |
| *K.pneumoniae* | 10^5^ | 92281.85 | 92.28 |
| *K.pneumoniae* | 10^6^ | 1083636.74 | 108.36 |
| *K.pneumoniae* | 10^7^ | 11019227.84 | 110.19 |
| *K.pneumoniae* | 10^8^ | 90447246.24 | 90.45 |
| *C.albicans* | 10^3^ | 1085.22 | 108.52 |
| *C.albicans* | 10^4^ | 10567.05 | 105.67 |
| *C.albicans* | 10^5^ | 93560.02 | 93.56 |
| *C.albicans* | 10^6^ | 983028.43 | 98.30 |
| *C.albicans* | 10^7^ | 9421475.93 | 94.21 |
| *C.albicans* | 10^8^ | 101855522.62 | 101.86 |

**References**

1. Aizitiaili M, Jiang Y, Jiang L, Yuan X, Jin K, Chen H, Zhang L, Qu X (2021) Programmable Engineering of DNA-AuNP Encoders Integrated Multimodal Coupled Analysis for Precision Discrimination of Multiple Metal Ions. Nano Lett 21(5):2141-2148. <https://doi.org/10.1021/acs.nanolett.0c04887>
2. Pei H, Li J, Lv M, Wang J, Gao J, Lu J, Li Y, Huang Q, Hu J, Fan C (2012) A Graphene-Based Sensor Array for High-Precision and Adaptive Target Identification with Ensemble Aptamers. J. Am. Chem Soc 134(33):13843-13849. <https://doi.org/10.1021/ja305814u>
3. Wei X, Wang Y, Zhao Y, Chen Z (2017) Colorimetric sensor array for protein discrimination based on different DNA chain length-dependent gold nanoparticles aggregation. Biosens Bioelectron 97:332-337. [https://doi.org/10.1016/j.bios.2017.06.020](https://doi.org/https://doi.org/10.1016/j.bios.2017.06.020)
4. Xu S, Wu Y, Sun X, Wang Z, Luo X (2017) A multicoloured Au NCs based cross-reactive sensor array for discrimination of multiple proteins. J Mater Chem B 5(22):4207-4213. <https://doi.org/10.1039/c7tb00367f>
5. Yang X, Li J, Pei H, Li D, Zhao Y, Gao J, Lu J, Shi J, Fan C, Huang Q (2013) Pattern recognition analysis of proteins using DNA-decorated catalytic gold nanoparticles. Small 9(17):2844-2849. <https://doi.org/10.1002/smll.201202772>
6. Jurs P C, Bakken G A, McClelland H E (2000) Computational methods for the analysis of chemical sensor array data from volatile analytes. Chem Rev 100(7):2649-2678. <https://doi.org/10.1021/cr9800964>
7. Zheng L, Qi P, Zhang D (2018) A simple, rapid and cost-effective colorimetric assay based on the 4-mercaptophenylboronic acid functionalized silver nanoparticles for bacteria monitoring. Sens Actuators B Chem 260:983-989. [https://doi.org/10.1016/j.snb.2018.01.115](https://doi.org/https://doi.org/10.1016/j.snb.2018.01.115)
8. Bordbar M M, Tashkhourian J, Tavassoli A, Bahramali E, Hemmateenejad B (2020) Ultrafast detection of infectious bacteria using optoelectronic nose based on metallic nanoparticles. Sens Actuators B Chem 319:128262. <https://doi.org/10.1016/j.snb.2020.128262>
9. Wang H, Zhou Y, Jiang X, Sun B, Zhu Y, Wang H, Su Y, He Y (2015) Simultaneous Capture, Detection, and Inactivation of Bacteria as Enabled by a Surface-Enhanced Raman Scattering Multifunctional Chip. Angew Chem Int Ed 54(17):5132-5136. <https://doi.org/10.1002/anie.201412294>
10. Huang L, Sun D-W, Wu Z, Pu H, Wei Q (2021) Reproducible, shelf-stable, and bioaffinity SERS nanotags inspired by multivariate polyphenolic chemistry for bacterial identification. Anal Chim Acta 1167:338570. <https://doi.org/10.1016/j.aca.2021.338570>
11. Besant J D, Das J, Sargent E H, Kelley S O (2013) Proximal Bacterial Lysis and Detection in Nanoliter Wells Using Electrochemistry. ACS Nano 7(9):8183-8189. <https://doi.org/10.1021/nn4035298>
12. Varshney M, Li Y (2007) Interdigitated array microelectrode based impedance biosensor coupled with magnetic nanoparticle–antibody conjugates for detection of Escherichia coli O157:H7 in food samples. Biosens Bioelectron 22(11):2408-2414. <https://doi.org/10.1016/j.bios.2006.08.030>
13. Xu M, Wang R, Li Y (2016) Rapid detection of Escherichia coli O157:H7 and Salmonella Typhimurium in foods using an electrochemical immunosensor based on screen-printed interdigitated microelectrode and immunomagnetic separation. Talanta 148:200-208. <https://doi.org/10.1016/j.talanta.2015.10.082>
14. Mo X, Wu Z, Huang J, Zhao G, Dou W (2019) A sensitive and regenerative electrochemical immunosensor for quantitative detection of Escherichia coli O157:H7 based on stable polyaniline coated screen-printed carbon electrode and rGO-NR-Au@Pt. Anal Methods 11(11):1475-1482. <https://doi.org/10.1039/C8AY02594K>
15. Yu M, Wang H, Fu F, Li L, Li J, Li G, Song Y, Swihart M T, Song E (2017) Dual-Recognition Förster Resonance Energy Transfer Based Platform for One-Step Sensitive Detection of Pathogenic Bacteria Using Fluorescent Vancomycin–Gold Nanoclusters and Aptamer–Gold Nanoparticles. Anal Chem 89(7):4085-4090. <https://doi.org/10.1021/acs.analchem.6b04958>
16. Shen Y, Lei F, Meng T, Li C, Yang Z, Huang J, Song F, Wan Y (2021) Gold nanoparticles-mediated fluorescent chemical nose sensor for pathogenic diagnosis and phenotype. J Mol Recognit 34(11):e2919. <https://doi.org/10.1002/jmr.2919>
